# Supported Gold Nanoparticles as Catalysts for the Oxidation of Alcohols and Alkanes

**DOI:** 10.3389/fchem.2019.00702

**Published:** 2019-11-05

**Authors:** Sónia A. C. Carabineiro

**Affiliations:** Centro de Química Estrutural, Instituto Superior Técnico, Universidade de Lisboa, Lisbon, Portugal

**Keywords:** gold, nanoparticles, carbon supports, metal oxides, alcohol, oxidation

## Abstract

Supporting gold nanoparticles have shown to be extremely active for many industrially important reactions, including oxidations. Two representative examples are the oxidation of alcohols and alkanes, that are substrates of industrial interest, but whose oxidation is still challenging. This review deals with these reactions, giving an insight of the first studies performed by gold based catalysts in these reactions and the most recent developments in the field.

## Introduction

Heterogeneous catalysis by gold nanoparticles is now a “hot topic,” as it can have applications in several industrially and environmentally important oxidation reactions (Thompson, 1998, 1999, 2006; Bond and Thompson, 1999, 2000; Sanchez et al., 1999; Haruta and Daté, 2001; Bond, 2002; Haruta, 2002, 2003, 2004, 2005; Ma et al., 2003; Corti et al., 2005; Bond et al., 2006; Hashmi and Hutchings, 2006; Carabineiro and Thompson, 2007, 2010; Corma and Garcia, 2008; Della Pina et al., 2008a, 2012; Hashmi and Rudolph, 2008; Song et al., 2010; Della Pina and Falletta, 2011; Pradal et al., 2011; Tsukuda et al., 2011; Mallat and Baiker, 2012; Rudolph and Hashmi, 2012; Takei et al., 2012; Liu X. Y. et al., 2013; Zhang and Ding, 2013; Majdalawieh et al., 2014; Takale et al., 2014; Alex and Tiwari, 2015; Biener et al., 2015; Freakley et al., 2015; Ishida et al., 2016; Pflasterer and Hashmi, 2016; Fang et al., 2017; Scurrell, 2017; Shahzad et al., 2017; Kim, 2018; Saldan et al., 2018; Zhao and Jin, 2018). Catalytic oxidations can be classified into two types (Sokolovskii, 1990): *complete (or total) oxidation*, used for catalytic destruction of various toxic compounds, and *selective oxidation*, used for organic compounds in fine chemistry, aiming at the synthesis of desired chemical products. Gold catalysts proved to be efficient for both types of oxidations (as shown on references cited above), including oxidation of CO, hydrogen production by water-gas shift (WGS), hydrogen purification by selective oxidation of CO in the presence of H_2_ (preferential oxidation, PROX), oxidative decomposition of volatile organic compounds (VOCs), selective oxidation of alcohols, hydrocarbons and sugars, among many others.

Gold nanoparticles also showed to be very good catalysts for the synthesis of fine chemicals, especially in selective oxidation (Biella et al., 2002a,b; Carrettin et al., 2002; Abad et al., 2005; Della Pina et al., 2008b, 2012; Hereijgers and Weckhuysen, 2009; Choudhary and Dumbre, 2011; Wu et al., 2011a,b,c; Biradar and Asefa, 2012; Xie et al., 2012; Wang H. et al., 2013; Sharma et al., 2016; Giorgi et al., 2017). This increasing interest is linked to environmental issues related with the need of more efficient processes and with new methods for synthesis of nanoparticles. As widely known in catalysis by gold, the need of small sized nanoparticles is mandatory (see references cited in the first paragraph). Gold can be a quite efficient catalyst, allowing high activities and selectivities, in some cases in solvent-free conditions, and without the use for harsh conditions (powerful oxidants, high temperatures and pressures), allowing mild conditions to be used (Biella et al., 2002a, 2003c; Pattrick et al., 2004; Moreno et al., 2013; Liu et al., 2014a; Villa et al., 2015b; Giorgi et al., 2017; Zhao and Jin, 2018).

Selective oxidation processes are used to activate raw materials and transform them into useful products and chemical intermediates (Carabineiro and Thompson, 2007, 2010). Some examples of such raw materials are alkanes and alcohols, which are of industrial interest, but are often characterized by low conversions and the formation of unwanted by-products. This review paper refers to the oxidations of those substrates, referring the first studies performed with gold based catalysts and the latest developments achieved so far, and it also includes a brief introduction about the main methods used for the preparation of this kind of catalysts.

## Gold Catalyst Preparation Methods

There are several techniques described in the literature to obtain well-dispersed gold nanoparticles on several supports. The most common, leading to efficient catalysts, are referred below.

### Sol-Immobilization (COL)

Colloidal Au can be synthesized in solution in the presence of an excess of stabilizing (capping) agents/ligands or surfactants (which can be as thiols, amines, polymers, phosphines, etc). This provides control of the size and shape of the formed nanoparticles, preventing them from agglomerating. Colloids prepared by reduction of chloroauric acid by citric acid, NaBH_4_, or other reducing agents, can be used to prepare gold on carbon or oxide supports, by deposition from the colloid, to give good dispersions of gold (Carabineiro and Thompson, 2010). The 1% Au/carbon prepared by this method by Rossi's group (Bianchi et al., 2000, 2003; Porta et al., 2000, 2002; Biella et al., 2002a,b, 2003a,b; Porta and Rossi, 2003; Comotti et al., 2004, 2005; Beltrame et al., 2006; Della Pina et al., 2007, 2008a,b, 2012; Chen et al., 2008) was distributed by the World Gold Council as a reference catalyst for the scientific community (World-Gold-Council, 2003). Other authors, including Carabineiro and co-authors, also successfully used this method to prepare active Au/carbon materials (Onal et al., 2004; Demirel-Gulen et al., 2005; Demirel et al., 2007b,c; Li B. D. et al., 2009; Zhu et al., 2010; Rodrigues et al., 2011, 2012a,b; Carabineiro et al., 2013; Ribeiro et al., 2017a,b; Tofighi et al., 2017).

Usually, a stabilizing agent is used in excess in order to effectively stabilize the nanoparticles. Then the colloids are deposited on the surface of the support to synthesize a heterogeneous catalyst. Thus, the stabilizing agent present in the colloidal solution might also form bonds with the support, and that can be detrimental as it might (partially) block the active metal sites (Donoeva and De Jongh, 2018). Also, the presence of stabilizing agents on the surface makes it more complicated to interpret catalytic results, since one does not know the effects they might have on the reaction (Niu and Li, 2014). Polyvinylpyrrolidone (PVP) is one of the most commonly used stabilizing agents (Onal et al., 2004; Demirel-Gulen et al., 2005; Demirel et al., 2007b,c; Shi et al., 2010; Wu J. et al., 2010; Zhu et al., 2010; Rodrigues et al., 2011, 2012b; Behera and Ram, 2012; Prati and Villa, 2012; Carabineiro et al., 2013; Koczkur et al., 2015; Du et al., 2016; Louie et al., 2017; Ribeiro et al., 2017a,b; Tofighi et al., 2017). Thus, the, removal of these compounds, used in the preparation, is of crucial importance. Usually, they are removed by decomposition at ~300°C (Onal et al., 2004; Demirel-Gulen et al., 2005; Demirel et al., 2007b,c; Zhu et al., 2010; Rodrigues et al., 2011, 2012b; Carabineiro et al., 2013; Ribeiro et al., 2017a,b; Tofighi et al., 2017). However, UV light, ozone and solvothermal treatments can also be used (Niu and Li, 2014). The progresses on this method have been recently reviewed (Prati and Villa, 2014).

### Impregnation (IMP) and Double Impregnation (DIM)

Impregnation (IMP) is the classical method used to prepare supported metal catalysts and consists of simply impregnating a support with a solution of a metal salt. This usually involves suspending the support in a larger volume of solution, from which the solvent is then removed. An alternative variation is the so-called incipient wetness (IW) technique, in which the pores of the support are filled with the solution.

Common gold precursors like chloroauric acid (HAuCl_4_) or auric chloride (AuCl_3_ or Au_2_Cl_6_) are usually used but complex salts, such as potassium aurocyanide (KAu(CN)_2_) and the ethylenediamine complex [Au(en)_2_]Cl_3_, are also alternatives. Traditional supports are silica, alumina and magnesia, but titania, alumina, boehmite (AlO(OH)), ferric oxide (α-Fe_2_O_3_) and magnesium hydroxide can also be used (Bond and Thompson, 1999). After drying, the precursor needs calcination at temperatures as high as 800°C. Reduction by hydrogen at 250°C, aqueous oxalic acid at 40°C or aqueous magnesium citrate (Bond and Thompson, 1999) is also needed.

**Figure 1:**
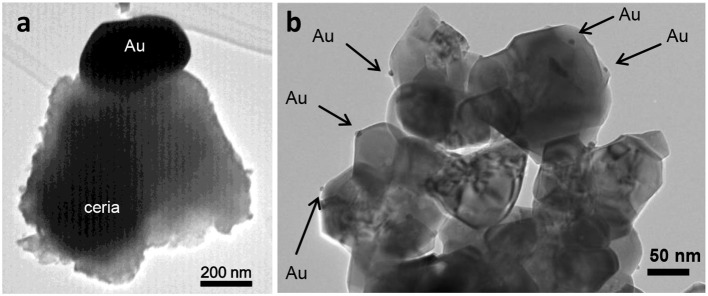
TEM images of Au/CeO_2_ prepared by traditional impregnation **(a)** and double impregnation **(b)**. Adapted from Carabineiro et al. (2010e,g), Copyright (2010), with permission from Elsevier.

Conventional impregnation techniques are not so adequate for gold catalysts as they result in large gold particles, which are more likely to be inactive (Bamwenda et al., 1997; Bond and Thompson, 1999; Lee and Gavriilidis, 2002; Carabineiro and Thompson, 2007, 2010; Carabineiro et al., 2010g; Santos et al., 2010). Moreover, it is difficult to obtain high dispersions of gold. Also, during calcination, the gold particles experience severe agglomeration as HAuCl_4_ interacts only weakly with the support (Haruta, 1997; Lee and Gavriilidis, 2002; Meyer et al., 2004; Carabineiro and Thompson, 2007). An example is displayed in Figure 1a. A transmission electron microscopy (TEM) image shows a 400 nm particle of Au on a ceria support.

The presence of chloride is detrimental, since it increases the mobility of Au on the support, leading to the sintering of gold particles (Oh et al., 2002; Kung et al., 2003; Bond et al., 2006; Carabineiro and Thompson, 2007, 2010; Carabineiro et al., 2010g). Gold and chloride ions combine to form bridges, favoring the growth of the particles upon heating (Hargittai et al., 2001; Schulz and Hargittai, 2001). The early use of classical impregnation techniques was the main reason why Au was thought for so long to be inactive for catalysts, in comparison with other noble metals, like Pt and Pd.

However, Datye and co-workers reported an alternative impregnation method for supporting gold on alumina using HAuCl_4_ (Xu et al., 2003). Since impregnation under acidic conditions leads to poor dispersion of Au and the resulting catalysts are not very active, a two-step procedure has been developed: In the first step, gold chloride was adsorbed on alumina from an acidified solution. After washing off the excess gold precursor, the solid was treated with strong base to convert the chloride to an adsorbed hydroxide. Drying and calcining at 400°C yielded a catalyst with gold particles having an average diameter of 2.4 nm, with good activity, and stability to hydrothermal sintering. This new method is a successful impregnation preparative route for gold catalysts, allowing them to have durability at least until 600°C, since no chloride is present.

Also Bowker et al. refer the use of a double impregnation method (DIM) that removes chloride (Soares et al., 2006; Bowker et al., 2007). Briefly, this method consists in impregnating the support with an aqueous solution of the gold precursor (HAuCl_4_) and then with a solution of Na_2_CO_3_, under constant ultrasonic stirring, followed by washing with water and drying in an oven overnight at 120°C. These authors used this method to prepare Au/TiO_2_ materials, and state that it represents an environmentally and economically favorable route to the production of highly active gold catalysts. This method has been used with great success for other supports, such as metal oxides and carbon materials by Carabineiro et al. (2010a,b,c,e,f, 2011a,b, 2012a,b, 2013, 2016); Bastos et al. (2012); Rodrigues et al. (2012b); Silva C. G. et al. (2014); Soria et al. (2014); Pérez et al. (2016). One example is shown in Figure 1b, where much smaller and better dispersed gold nanoparticles are seen on ceria. However, the sol immobilization method is better for carbon catalysts (Rodrigues et al., 2012b; Carabineiro et al., 2013), as stated above.

### Co-Precipitation (CP)

This is one of the simplest ways to prepare gold catalysts and it was one of the first to be used (Haruta et al., 1987; Bond et al., 2006; Carabineiro and Thompson, 2007). Its discovery was by serendipity in 1987: Haruta's group mixed HAuCl_4_, iron nitrate and sodium carbonate, and produced a Au/Fe_2_O_3_ composite with low Au nanoparticle size (Haruta et al., 1987). Back then, classical impregnation was the method often used to prepare PGM catalysts, and it did not work when used for gold, as said above. Haruta's Au/Fe_2_O_3_ material showed unprecedented catalytic activity for CO (and hydrogen) oxidation, being active at sub-ambient temperatures, a range of temperatures never reached before for this reaction (Haruta et al., 1987) [as gold exhibits low affinity for CO adsorption below 200°C (Vigneron and Caps, 2016)].

The method is still widely applied (Sreethawong et al., 2011; Liu R. et al., 2013; Tran Thi Minh et al., 2013; Wang H. et al., 2013; He et al., 2016; Ilieva et al., 2016; Liu et al., 2016; Mishra et al., 2016; Li et al., 2018; Patil et al., 2018). It consists on having an aqueous solution of HAuCl_4_ and water-soluble metal salts, such as a nitrate, being poured into an aqueous alkaline solution (Na_2_CO_3_ and/or NH_4_OH) and stirred for a few minutes. The two hydroxides (or hydrated oxides) are then precipitated simultaneously. After aging for about 1 h, the precipitates are washed several times with water and filtered. The hydroxide and/or carbonate mixture is dried overnight and calcined to obtain powder catalysts (Lee et al., 2001; Seker and Gulari, 2002; Carabineiro and Thompson, 2007). Then a reduction step is necessary. It is possible that some catalysts prepared by this method contain a significant concentration of Na^+^ and Cl^−^ ions, if a metal chloride is used as precursor. Both can act as a catalyst poison (Haruta, 1997; Bond and Thompson, 1999; Oh et al., 2002; Bond et al., 2006; Carabineiro and Thompson, 2007, 2010; Carabineiro et al., 2010g; Zhang et al., 2013). However, alkali have also been reported to enhance the activity of Au catalysts (Broqvist et al., 2004; Huang et al., 2011a,b; Li Y. et al., 2011; Nepak and Srinivas, 2015; Ribeiro et al., 2017a,b; Rodriguez et al., 2018). An understanding of the effect of residual Na would be an excellent way to advance gold research (Veith, 2016). The applicability of this technique is limited to metal hydroxides or carbonates that can be co-precipitated with Au(OH)_3_.

### Deposition Precipitation (DP)

This method is convenient and can be used for producing commercial gold supported catalysts (Haruta, 2003; Carabineiro and Thompson, 2007) and applied to the widest range of different support materials (Wolf and Schüth, 2002). It was discovered also by Haruta's group in 1991 (Tsubota et al., 1991) and is still widely used (Moreau et al., 2005; Sangeetha et al., 2009; Wei et al., 2010; Tran et al., 2011; Sanada et al., 2013; Soria et al., 2014; Zahoranova et al., 2015; Zamaro et al., 2015; Pérez et al., 2016; Priecel et al., 2016; Carabineiro et al., 2017; Chen B. B. et al., 2017; Kyriakou et al., 2017; Martins et al., 2017; Rodrigues et al., 2017a,b; Vourros et al., 2017; Yu et al., 2017; Jin et al., 2018). The precursor to the active species is brought out of solution in the presence of a suspension of the support, usually by raising the pH in order to precipitate a hydroxide. The surface of the support acts as nucleating agent, and most of the active precursor ends up being attached to the support. After the pH of an aqueous solution of HAuCl_4_ is adjusted with NaOH, to a fixed point in the range of 6–10, a metal oxide support can be immersed in the solution. The partially hydrolysed species [Au(OH)_n_Cl_4−n_]^−^ (n = 1–3) then react with the surface of the support. Aging for about 1 h results in the deposition of Au(OH)_3_, exclusively on the surface of the metal oxide support, if the concentration and temperature are properly chosen (Haruta, 1997; Bond and Thompson, 1999; Carabineiro and Thompson, 2007).

**Figure 2:**
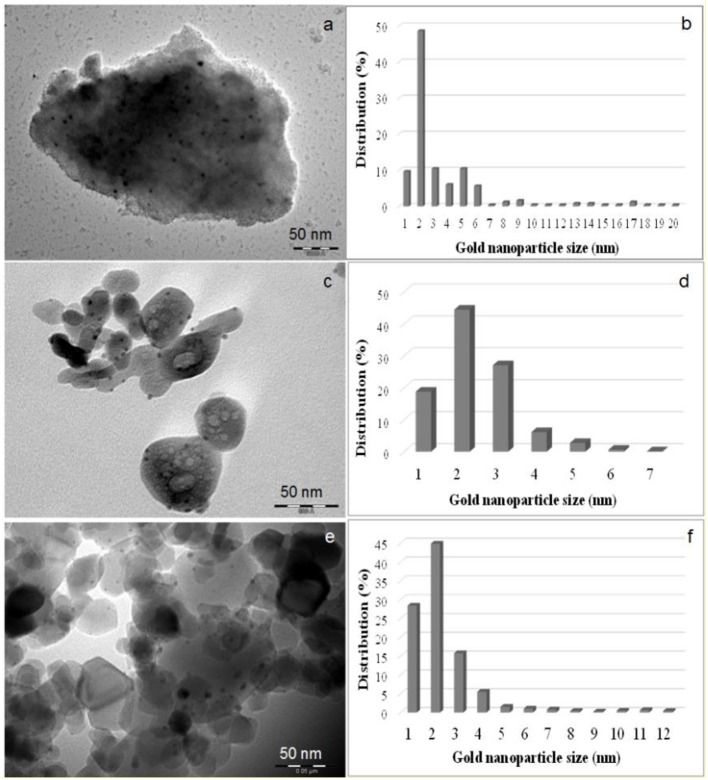
TEM images of Au/Al_2_O_3_ **(a)**, Au/Fe_2_O_3_ **(c)**, and Au/TiO_2_ **(e)** along with the corresponding size distribution histograms of gold nanoparticles **(b,d,f)**. Used with permission from Martins et al. (2017). Copyright (2017) Wiley.

The influence of the pH on the particle size of Au is remarkable, as above pH 6, the main species of Au in solution are transformed from ${\text{AuCl}}_{4}^{-}$ to [Au(OH)_n_Cl_4−n_]^−^ (n = 1–3), and the mean particle diameters of Au in the calcined catalysts become smaller than 4 nm (Haruta, 1997). Several authors claim that a pH ranging from 7 to 8 is preferable depending on the oxide support (Wolf and Schüth, 2002; Kung et al., 2003; Wang et al., 2003; Zahoranova et al., 2015), since at this pH, the value of *n* is close to 3, and at lower values of pH, there is less hydrolysis of the Au–Cl bond. However, Bond and co-workers (Moreau et al., 2005) showed that pH 9 was the optimum value to be reached during deposition precipitation for Au/TiO_2_ catalysts, and that value has been used by Carabineiro and co-authors to prepare different Au/oxide catalysts (Soria et al., 2014; Pérez et al., 2016; Carabineiro et al., 2017; Kyriakou et al., 2017; Martins et al., 2017; Rodrigues et al., 2017a,b; Vourros et al., 2017). Some examples of catalysts thus obtained are shown in Figure 2. At that pH, the main species in solution are anionic Au complexes, with almost no chloride, while at lower values, the Au complexes contain chloride, Au particles are larger, and activities lower. However, the optimum pH range for precipitation that also assures an efficient metal utilization (>90%) depends on the isoelectric point of the supporting material (Prati and Villa, 2012).

An alternative method for adjusting the pH is to use urea (Bond and Thompson, 1999; Dobrosz et al., 2005; Zanella et al., 2005; Gluhoi and Nieuwenhuys, 2007; Hugon et al., 2010). This consists in the slow decomposition of urea in the solution. Hydroxyl ions are generated slowly and uniformly throughout the liquid phase, and their concentration is always low because they are consumed almost as soon as they are formed. Louis and co-workers found that the use of urea yielded the same gold particle sizes as those obtained using NaOH (2–3 nm), and no sodium poison was introduced (Zanella et al., 2005).

A constraint of the deposition-precipitation is that it is not very suitable for activated carbon (Prati and Martra, 1999; Haruta, 2003; Carabineiro and Thompson, 2007, 2010; Prati and Villa, 2012) or zeolites (Lin and Wan, 2003), due to their high isoelectric point [although some recent publications showed better results for these materials, especially after an acidic treatment (Cardenas-Lizana et al., 2015; Behravesh et al., 2017)]. Deposition-precipitation has the advantage over co-precipitation in that all of the active component remains on the surface of the support and none is buried within it (Bond and Thompson, 1999; Wang et al., 2003; Carabineiro and Thompson, 2007). Also, it gives narrower particle size distributions, but it is recommended that the support should have a surface area of at least 50 m^2^/g (Bond and Thompson, 1999; Carabineiro and Thompson, 2007).

### Liquid-Phase Reductive Deposition (LPRD)

This method was first used by Sunagawa et al. and consists of mixing a solution of the gold precursor (HAuCl_4_) with a solution of NaOH with stirring at room temperature (Sunagawa et al., 2008). The resulting solution is aged for 24 h, in the dark, at room temperature, to complete the hydroxylation. Then, the appropriate amount of support is added to the solution and, after ultrasonic dispersion for 30 min, the suspension is aged in the oven at 100°C overnight. The resulting solid is washed repeatedly with distilled water for chloride removal and dried in the oven at 100°C, overnight.

The selective reductive deposition is performed by adsorption of the gold ions onto the surfaces where the reduction takes place. Thus, the initial adsorption is the key feature of this technique, and the key points are precise control of the metal complex by adjusting solute conditions, such as composition and structure of the metal complex; storing of the suspension until the equilibrium composition is attained, and aging the suspension at a controlled temperature. This method was later used by Carabineiro and co-authors to successfully prepare Au nanoparticles supported on various carriers (Carabineiro et al., 2010c,g, 2012b; Santos et al., 2010, 2014; Rodrigues et al., 2012b; Soria et al., 2014; Pérez et al., 2016).

### Ion-Exchange

In this method, ions on the surface of the support are replaced by gold ions. The procedure is especially effective with zeolites. But the introduction of catalytically active species into the cavities of these materials, as opposed to placing them on their external surface, presents certain difficulties, namely the lack of suitable cations or cationic complexes (Bond and Thompson, 1999). Nevertheless, different kinds of Au/zeolite systems have been prepared this way (Kang and Wan, 1995, 1997; Horvath et al., 2001; Chen et al., 2005; Magadzu et al., 2007; Tuzovskaya et al., 2007; Bogdanchikova et al., 2008; Sierraalta et al., 2008; Qi et al., 2012; Sanada et al., 2013; Zeng et al., 2014; Zamaro et al., 2015; Emayavaramban et al., 2016b; Chen B. B. et al., 2017).

Pitchon and co-workers have developed a novel method for preparing supported gold catalysts based on the direct anionic exchange (DAE) of the gold species with the hydroxyl groups of the support (Ivanova et al., 2004, 2006a,b; Dobrosz et al., 2005; Dobrosz-Gomez et al., 2009; Azizi et al., 2010; Liao et al., 2012). An aqueous solution of HAuCl_4_ is added to the support, heated to 70°C and aged for 1 h. The slurry is then filtered, washed with warm water, dried in an oven at 120°C, overnight, and calcined in air at 300°C, for 4 h. In order to completely remove the chloride ions, these authors take a fraction of the catalyst after drying and wash it with a concentrated solution of ammonia. However, this may be a dangerous procedure, since gold oxide and ammonia sometimes produce fulminating gold, which is explosive (Fisher, 2003; Bond et al., 2006; Carabineiro and Thompson, 2007, 2010; Steinhauser et al., 2008).

### Photochemical Deposition (PD)

This method allows metal deposition over semiconductor materials, with simultaneous reduction of metal ions by the electrons of the conduction band (Carabineiro et al., 2010d). This process can be enhanced by addition of “sacrificial electron donors” (such as formaldehyde, methanol or 2-propanol) that can supply an almost unlimited amount of electrons. Photodeposition takes place at, or near, the photoexcited sites, leading to an enhanced dispersion. The gold precursor is usually dissolved in water and a sacrificial electron donor, mixed with the support, sonicated and photodeposited using a UV lamp.

This method has been used for preparation of gold catalysts, mostly on TiO_2_ (Wang et al., 1998, 2011; Chan and Barteau, 2005; He et al., 2006; Ruvarac-Bugarcic et al., 2008; Hidalgo et al., 2009, 2011; Sangeetha et al., 2009; Yang et al., 2009; Yogi et al., 2009; Carabineiro et al., 2010d; Kydd et al., 2010; Murcia-Mesa et al., 2010; Tanaka et al., 2012, 2014; Mei et al., 2013; Song et al., 2015; Negishi et al., 2017). Carabineiro et al. used it for the first time to deposit Au on ZnO supports (Carabineiro et al., 2010c,d). Other authors also prepared Au/ZnO (Naknam et al., 2009; Fernando et al., 2016; Wang X. W. et al., 2016; Andrade et al., 2017), and Au on other supports, such as CeO_2_, C_3_N_4_, CdS, ZrO_2_, CuCrO_2_, quartz, etc. (Boitsova et al., 1999; Kydd et al., 2010; Kominami et al., 2011; Chiu et al., 2014; Jiang et al., 2015; Singh and Pal, 2015; Xue et al., 2015).

### Ultrasonication (US)

**Figure 3:**
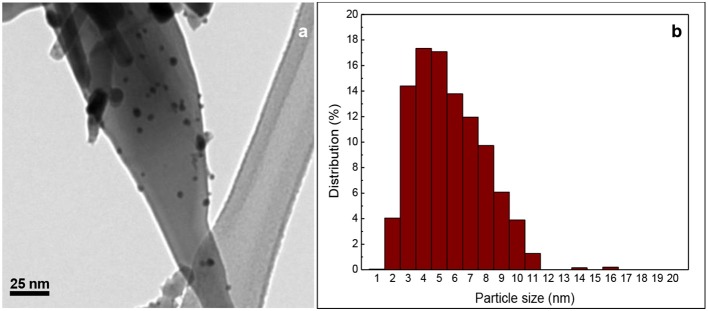
**(a)** TEM image of Au/ZnO_CVD_ prepared by US (support prepared by chemical vapor deposition) and **(b)** Au nanoparticle size distribution. Reprinted from Carabineiro et al. (2010c), Copyright (2010), with permission from Elsevier.

This method is similar to PD but without photodeposition; instead the sample is sonicated during 8 h (Carabineiro et al., 2010c). The procedure was a serendipitous discovery made by Carabineiro et al. when attempting to prepare gold on zinc oxide by PD [the ZnO support used was prepared by chemical vapor deposition, ZnO_CVD_, according to a previously described procedure (Bacsa et al., 2009)]. The Au/ZnO sample was supposed to be sonicated for 30 min (then following the photodeposition step), but it was left in the fume hood and sonicated for 8 h. After that time, the mixture showed a deep purple color, similar to the samples prepared by other methods. So, it was washed and dried normally. The obtained material (Au/ZnO_CVD_ US) was tested for CO oxidation and turned out to be the most active catalyst of the study (Carabineiro et al., 2010c). A TEM image with the respective histogram of size distribution is shown in Figure 3. US was also used by the same authors to prepared Au on Fe_2_O_3_ (Carabineiro et al., 2012a), MgO (Carabineiro et al., 2011b), CuO, NiO, La_2_O_3_, and Y_2_O_3_ materials (Carabineiro et al., 2011a), however the results obtained were not as good as those on Au/ZnO (Carabineiro et al., 2010c).

### Vapor-Phase Methods and Grafting

These methods are similar, differing only if a solvent is present or not. In the vapor-phase method (*Chemical Vapor Deposition*), a stream of a volatile compound of gold is transported onto a high area support by an inert gas and it reacts chemically with the surface of the support to form a precursor to the active species (Haruta, 1997; Carabineiro and Thompson, 2007, 2010). The most widely used gold precursor is AuCl_3_ or HAuCl_4_, but other substances have also been used, mainly to prevent chloride contamination. Gold particles have successfully been incorporated into TiO_2_, WO_3_, and MoO_3_ thin films using a single step process by this technique (Manna et al., 2016). Recently, it was shown that chemical vapor deposition can be used to synthesize gold nanocrystals with various morphologies, such as prisms, icosahedrons, and 5-fold twinned decahedrons on silicon substrates (Manna et al., 2016). The advantage of this method is that high-quality anisotropic crystals of gold can be produced without the need for surfactants or templates.

In *Physical Vapor Deposition*, gold is vapourized from a target under vacuum and deposited on an oxide support or carbon under high vacuum conditions (Carabineiro and Thompson, 2010). The 3M company in Minnesota (USA) has found that very active gold nanocatalysts can be prepared this way on a wide range of supports, including some that are water soluble or not suitable for deposition-precipitation, like SiO_2_ (Brey et al., 2005; Brady et al., 2006). This method is low-cost, has great reproducibility, does not need washing and thermal treatment steps, as do those materials resulting from solution preparation methods, and has no toxicity hazards. Recently, gold nanoparticles with high thermal stability (up to 600°C) were supported on Al_2_O_3_, using this technique (Smirnov et al., 2016).

In the *grafting* method, a gold complex in solution reacts with the surface of a support, forming species convertible to a catalytically active form. Thus, gold phosphine complexes have been grafted onto the surface for a number of freshly precipitated wet hydroxides (Yuan et al., 1997; Kozlova et al., 1998, 1999; Bond and Thompson, 1999; Kozlov et al., 1999, 2000; Olea and Iwasawa, 2004; Carabineiro and Thompson, 2007), since they have many surface –OH groups reactive to the Au compounds. Vacuum-drying at room temperature and temperature-programmed calcination in air follows, causing simultaneous transformation of both precursors to gold particles and oxides, respectively, under their chemical interactions by temperature-programmed calcination. Au–phosphine complexes are choice candidates for metal precursors because they thermally decompose to Au metal in a temperature range similar to that used for the transformation of wet metal hydroxides to oxides. Moreover, the phosphine ligands are expected to retard the growth to large Au metallic particles. Gold can be deposited on SiO_2_, MCM−41, SiO_2_-Al_2_O_3_, and activated carbon, as nanoparticles with high dispersion by the gas-phase grafting of an acetylacetonate complex of gold, while liquid-phase preparation methods are not that effective with these supports (Okumura et al., 2003).

### Bi- and Tri-Metallic Gold Catalysts

Since gold is already established on its own, more advances are always expected when it is combined with other metals, in order to increase its activity/selectivity (Carabineiro and Thompson, 2007). Gold-based bimetallic catalysts showed great potential for many important chemical transformation reactions, owing to their good activity and high selectivity under relatively mild conditions, in reactions such as selective oxidation, selective hydrogenation, C-C coupling and photocatalysis, as recently reviewed (Zhao and Jin, 2018). There are many cases in the literature of bimetallic gold catalysts prepared by several techniques, as shown in several recent reviews (Kharisov et al., 2009; Li B. D. et al., 2009; Shah et al., 2012; Zhao et al., 2013; Hutchings, 2014; Freakley et al., 2015; Villa et al., 2015c; Alshammari et al., 2016; Louis, 2016; Priecel et al., 2016; Zhang L. et al., 2016).

### Post-treatment and Storage

After preparing the gold-based catalyst, a variety of post-treatment conditions can be used, including calcination or reduction (Tsubota et al., 1998; Kozlov et al., 1999; Boccuzzi et al., 2001; Fu et al., 2003; Moreau et al., 2005; Bond et al., 2006; Ivanova et al., 2006a; Carabineiro and Thompson, 2007, 2010; Huang et al., 2011b; Carabineiro et al., 2012b; Ayastuy et al., 2016, 2017). It is worth to note that many catalysts are used effectively without any need of such treatments. In fact, there are times when reduction or calcination is even detrimental (Bond and Thompson, 2000; Moreau et al., 2005). The size of the gold particles can also influenced by the thermal treatment (Bond et al., 2006; Carabineiro and Thompson, 2007, 2010). Nevertheless, samples prepared by the sol-method described above, are often heat treated to decompose the organic scaffold (Onal et al., 2004; Demirel-Gulen et al., 2005; Demirel et al., 2007b,c; Li B. D. et al., 2009; Zhu et al., 2010; Rodrigues et al., 2011, 2012b; Ribeiro et al., 2017a,b; Tofighi et al., 2017).

It is recommended that “as prepared” samples are stored in a refrigerator at 0°C and that calcined catalysts should also be kept cold, and that, after drying, samples are kept in a vacuum desiccator in the dark, with reduction being performed immediately before use (Zanella and Louis, 2005; Lee et al., 2007; Wu et al., 2008; Raphulu et al., 2009; Carabineiro and Thompson, 2010; Wei et al., 2010; Tran et al., 2011).

## Selective Oxidation Using Gold Catalysts

Prati and Rossi's group studied the liquid phase oxidation of *several organic substrates* (alcohols, sugars, aldehydes, amines, imines, etc.), showing that Au/carbon was the preferred catalyst (Prati and Rossi, 1998; Prati and Martra, 1999; Bianchi et al., 2000, 2003, 2005; Porta et al., 2000, 2002; Biella et al., 2002a,b, 2003a,b,c; Porta and Rossi, 2003; Comotti et al., 2004, 2005; Porta and Prati, 2004; Prati and Porta, 2005; Beltrame et al., 2006; Della Pina et al., 2007, 2008a,b, 2012; Della Pina and Falletta, 2011; Prati and Villa, 2012, 2014; Prati et al., 2012, 2018; Wang D. et al., 2013; Villa et al., 2015b,c; Dimitratos et al., 2016; Jouve et al., 2018; Motta et al., 2018), compared to silica, alumina or titania (Porta et al., 2000, 2002). The method dealing with immobilization of colloidal particles (COL) was one of the best preparation procedures used (Prati and Martra, 1999; Villa et al., 2013b; Prati and Villa, 2014; Jouve et al., 2018), and narrow nanoparticle size distribution was obtained (around 3–6 nm). Carbon supports are naturally microporous thus providing a protection for the small Au nanoparticles, allowing to limit their diameter.

Gold catalysts also showed better resistance to deactivation and poisoning. These are limiting factors in liquid phase oxidation (Bond et al., 2006; Carabineiro and Thompson, 2007, 2010).

## Alcohol Oxidation

The liquid phase oxidation of alcohols is a good example of a selective oxidation reaction, important in both academia and industry, which is an interesting path for obtaining fine chemicals and intermediates (Besson and Gallezot, 2000; Sheldon and Van Bekkum, 2001; Mallat and Baiker, 2004; Enache et al., 2006; Dimitratos et al., 2012; Guo et al., 2014; Ranveer et al., 2015; Olenin et al., 2018; Torbina et al., 2018). It has been the subject of important research, due the need to use renewable biomass-derived feedstocks and replace toxic oxidants by more environmentally friendly ones. In the past, oxidation reactions were carried out with the use of strong oxidants, like KMnO_4_, Jones reagent (chromium trioxide in diluted sulfuric acid), pyridine dichromate and RuO_4_, which increased the costs and produced large amounts of toxic wastes (Zhao et al., 1998; Tojo and Fernández, 2006). A large decrease in the amounts of chemical waste and pollution can be obtained if those oxidants are replaced by “greener” ones (like molecular oxygen and H_2_O_2_) (Dimitratos et al., 2012).

Gold catalysts have successfully been used in the oxidation of alcohols, as shown by several reviews (Besson and Gallezot, 2000; Prati and Porta, 2005; Bond et al., 2006; Hashmi and Hutchings, 2006; Hutchings et al., 2006; Carabineiro and Thompson, 2007, 2010; Edwards et al., 2007; Ishida and Haruta, 2007; Fristrup et al., 2008; Della Pina and Falletta, 2011; Della Pina et al., 2012; Dimitratos et al., 2012; Takei et al., 2012; Hutchings, 2014; Freakley et al., 2015; Sharma et al., 2016). Gold based materials have also been successfully used in alcohol photooxidation (Nizova and Shulpin, 1992; Zhang et al., 2012; Yu et al., 2014; Luken et al., 2015; Chen et al., 2016; Chasse and Hallett-Tapley, 2018).

### Oxidation of Diols

**Scheme 1:**
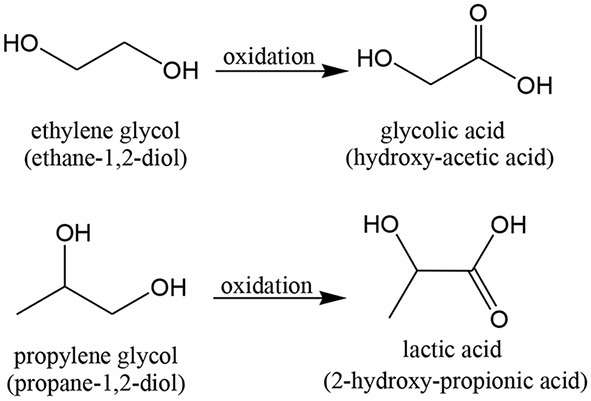
Examples of diols used in selective oxidation reactions and respective products.

The first report on the use of gold nanoparticles on carbon and alumina was released in 1998 by Prati and Rossi, referring to alcohol oxidation (Prati and Rossi, 1998). The materials were prepared by IMP and DP. In that work, ethane-1,2-diol and propane-1,2-diol were oxidized to the respective monoacids, in an alkaline (aqueous) solution, with high selectivity (Scheme 1). Gold was highly selective for the mono-oxidation of ethane-1,2-diol to glycolate, compared to Pt and Pd. For propane 1,2-diol, the gold catalyst allowed to obtain only lactate (total selectivity). Au catalysts also showed very good stability in recycling tests, higher than the Pt and Pd materials. These results are very important due to the industrial interest for glycolic acid and lactic acid. In fact, the usual chemical synthesis methods involves toxic and corrosive reagents, high-pressure equipment and alternative fermentation processes (used for lactic acid production), which show low productivity and complicated problems with purification (Bond et al., 2006; Carabineiro and Thompson, 2007).

Subsequent studies by the same group (Prati and Martra, 1999; Bianchi et al., 2000; Porta et al., 2000, 2002; Biella et al., 2003c; Porta and Rossi, 2003; Comotti et al., 2005) confirmed that Au/carbon was very active in the *selective oxidation of 1,2-diols to* α*-hydroxyacids*, in mild conditions, much better than mono-, di-, and tri-metallic Pd-, Pt-, and Bi-based catalytic systems, as Au showed more selectivity and more resistance to poisoning. Nevertheless, it requires strong alkaline conditions, which also enhanced selectivity. Au/carbon was also the most stable in recycling tests, without deactivation or leaching being observed.

### Oxidation of Polyalcohols

**Scheme 2:**
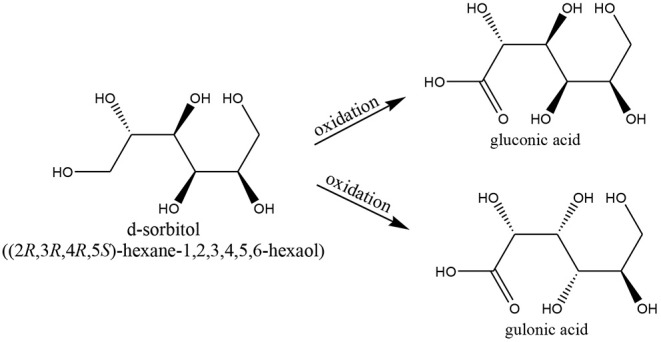
d-Sorbitol oxidation reactions.

Mixed Au–PGM/C were also tested by the same group in the selective oxidation of the polyalcohol *d-sorbitol* (Scheme 2) to gluconic and gulonic acids (Dimitratos and Prati, 2005; Dimitratos et al., 2005b; Prati and Porta, 2005). Bimetallic catalysts showed a resistance to poisoning and improved selectivity compared to monometallic. The addition of Au, after Pd or Pt being added and reduced, produced the best results (Dimitratos and Prati, 2005). Au and Au/Pt showed selectivities of 60% and 62%, respectively, to gluconate/gulonate (Dimitratos and Prati, 2005; Dimitratos et al., 2005b; Prati and Porta, 2005).

**Scheme 3:**
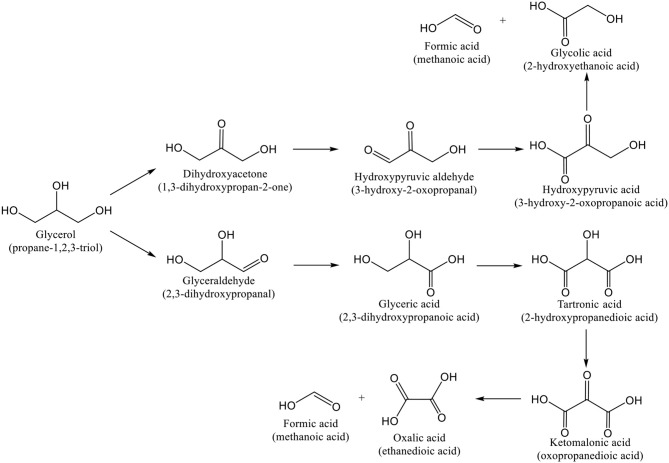
Reactions of glycerol oxidation under basic conditions (adapted with permission from Villa et al., 2015b). Copyright (2015) American Chemical Society.

The oxidation of *glycerol* is also a very important reaction. The glycerol molecule has many functionalizations, is obtainable from sustainable bio-sources, like sunflower crops and rapeseed, from which several products can be formed by oxidation (Scheme 3), and it is important that the process allows selectivity to distinct products aiming at making their use as chemical intermediates economically viable (Hutchings, 2005; Villa et al., 2015b; Prati and Villa, 2017). Glyceric acid and dihydroxyacetone (Scheme 3) can be used as chemical intermediates in the industry of fine chemistry, namely in pharmaceuticals (Zhou et al., 2008). To date, these molecules are commercially obtained using either expensive and polluting oxidation processes (like glyceraldehyde) or by microbial (incomplete) fermentation by *Gluconobacter oxidans* (like dihydroxyacetone) (Pagliaro et al., 2007, 2009; Zhou et al., 2008; Hu et al., 2010; Ciriminna et al., 2014). As glycerol has a high boiling point, its selective oxidation is often carried out using water as liquid medium and O_2_ as oxidant ((Porta and Prati, 2004); Carabineiro and Thompson, 2007).

Glyceraldehyde is the major product obtained from glycerol oxidation, using Pt or Pd catalysts on activated carbon, with a small amount of dihydroxyacetone (Garcia et al., 1995). The main disadvantage of catalysts that are based on these metals is that they tend to deactivate after some reaction time, due to poisoning by oxygen (Besson and Gallezot, 2000; Porta and Prati, 2004). Au catalysts are more resistant to oxygen poisoning compared to PGMs, permitting the use of high oxygen partial pressures (Prati and Rossi, 1998). However, they require the use of a basic medium to ensure a good conversion of glycerol (Carrettin et al., 2003; Hutchings et al., 2006; Zope et al., 2010; Villa et al., 2015b). Moreover, their activity (and selectivity) are also dependent of Au nanoparticle size which is also dependent on the method of preparation method and on the support.

Hutchings and co-workers published some studies dealing with glycerol oxidation in the liquid phase using Au/charcoal (Carrettin et al., 2002), Au/activated carbon (Carrettin et al., 2003) or Au/graphite (Carrettin et al., 2002, 2003, 2004). Before that, glyceraldehyde could be obtained with a selectivity of 70–80% using Pt catalysts (Garcia et al., 1995). However, Hutchings and co-workers (Carrettin et al., 2002, 2003, 2004; Hutchings et al., 2006) showed that glycerol oxidation could yield glyceric acid (Scheme 3) with high conversion and total selectivity, for 1% Au/charcoal or 1% Au/graphite catalysts, in mild conditions (60°C, 3 h, using water as solvent) (Carrettin et al., 2002). Without NaOH, Au/C was inactive and the formation of undesirable C-1 products (like formic acid, Scheme 3) was eliminated when NaOH was added (Carrettin et al., 2003). It was proposed that the base aided the initial dehydrogenation by abstraction of the H of the primary OH group of glycerol and, thus, allowing to overcome the rate limiting step of the oxidation (Carrettin et al., 2003).

Claus's group also investigated this reaction using gold catalysts on carbon supports [carbon black (Demirel-Gulen et al., 2005; Demirel et al., 2007a,b,c), activated carbon (Demirel et al., 2007a,b) and graphite (Demirel-Gulen et al., 2005)] and oxides [Al_2_O_3_ (Demirel-Gulen et al., 2005), MgO (Demirel-Gulen et al., 2005), TiO_2_ (Demirel-Gulen et al., 2005; Demirel et al., 2007a), and CeO_2_ (Demirel et al., 2007a)]. The oxide materials were prepared by DP using urea, while the carbon materials were prepared by COL. The carbon black gave better results than activated carbon or graphite (Demirel-Gulen et al., 2005; Demirel et al., 2007a,b,c). Results showed that the oxidation of glycerol is structure-sensitive reaction, as the selectivity to the glyceric acid product increased up to 75% with decreasing Au nanoparticle size (down to the best value of 3.7 nm) on carbon black (Demirel-Gulen et al., 2005). The selectivity to glyceric acid product was 40% for smaller Au nanoparticles (2.7 nm), but the glycolic acid selectivity increased from 15 to 36% (Demirel-Gulen et al., 2005). This showed that the nanoparticle particle size of the Au/carbon catalysts could play an important role in the reaction.

Porta and Prati studied these reactions on Au/carbon catalysts, namely activated carbon (Porta and Prati, 2004; Bianchi et al., 2005; Prati and Porta, 2005; Jouve et al., 2018) and graphite (Porta and Prati, 2004). Au on activated carbon was more active than Au on graphite. Well-dispersed nanoparticles on activated carbon of 6 nm, were not able to maintain the preliminary selectivity up to full conversion, but larger (>20 nm) nanoparticles were able to maintain constant selectivity during the reaction time (Porta and Prati, 2004). This showed that gold nanoparticle size was not the only issue, but other factors, such as preparation method (with COL being better than IMP), and temperature (since an increase in the temperature promoted glyceric acid oxidation to tartronic acid, Scheme 3) could also play an important role. 92% of selectivity was found at full conversion (Porta and Prati, 2004).

The same group also used gold on (multi-walled) carbon nanotubes (CNTs) (Prati et al., 2011, 2016) and carbon nanofibers (CNFs) (Prati et al., 2011; Wang D. et al., 2013; Villa et al., 2016). It was shown that the basicity of CNFs led to an activity increase, but the selectivity was mostly linked to the nature of the surface groups, as the selectivity to C-3 products was best for surfaces with basic and hydrophobic nature, but surfaces more hydrophilic led to an increase of the products of C–C bond cleavage (Prati et al., 2011).

Au nanoparticles were also supported on CNFs with different degrees of graphitization (Wang D. et al., 2013). The CNF surface containing a larger amount of ordered graphitic layers led to gold nanoparticles preferentially immobilized on the (111) plane, with more facet area. Higher C-3 product selectivity was found on the (111) surface, showing that larger Au nanoparticles were more selective toward C-3 products compared to the smaller ones.

Addition of nitrogen to CNFs also had positive results (Villa et al., 2016). Au nanoparticles trapped within N-functionalized CNFs were more efficient for glycerol oxidation and promoted selectivity for di-acid products, while Au nanoparticles trapped on the surface produce the molecule derived from C–C cleavage as a major by-product.

Nitrogen doped CNTs were also used (Prati et al., 2016). The introduction of nitrogen functionalities was performed by high temperature treatment (600°C) in the presence of NH_3_. The turnover frequency (TOF, moles of product per mol of Au catalyst per time) obtained for N-doped CNTs was 853 h^−1^, much higher than the 182 and 186 h^−1^ obtained for pristine and oxidized CNTs, respectively. The selectivity to glycerate was 68% for the N-doped material and 55% for the pristine sample (for 90% conversion).

Carabineiro and co-authors compared several metals (Pt, Pd, Ir, Rh, and Au) on activated carbon as catalysts for glycerol oxidation, showing, for the first time, that that Rh could be an active catalyst for this reaction, although having high sensitivity to oxygen poisoning, as other PGMs (Rodrigues et al., 2011). IMP and COL were used as preparation methods. Not surprisingly, IMP yielded an inactive Au material. However, the Au catalyst prepared by the COL exhibited a high activity with only 0.32% Au loading, reaching a full glycerol conversion in ~3 h, in the standard conditions tested (with ~60% selectivity to glyceraldehyde). In contrast, only 44% was achieved with the reference Au/C catalyst (supplied by the World Gold Council, consisting on 0.8% Au on carbon black).

The same authors also tested Au nanoparticles supported on (multi-walled) CNTs prepared by different methods (Rodrigues et al., 2012a,b). COL was the most suitable method, yielding 47% selectivity toward glyceric acid (Rodrigues et al., 2012a,b). This reaction was also studied on Au nanoparticles deposited on carbon xerogels with different mesopore sizes (prepared by condensation of resorcinol and formaldehyde at different values of pH) by the COL method (Rodrigues et al., 2012c). It was found that larger pores (20 nm) enhanced the oxidation toward dihydroxyacetone, whereas smaller pores (5 nm) favored the formation of glyceric acid (Scheme 3).

Prati and co-workers also tested Au on metal oxides (Al_2_O_3_, MgO, MgAl_2_O_4_ spinel) prepared by DP and COL (Bogdanchikova et al., 2017). In terms of materials prepared by DP, Au/Al_2_O_3_ was more active than Au/MgO, but for catalysts prepared by COL, Au/MgO was more active than Au/alumina. Au/MgAl_2_O_4_ spinel showed high activities, similar to materials prepared by both methods. The same group also used a weak basic anion resin as support for Au nanoparticles (Villa et al., 2010).

Those authors also studied mono- and bimetallic Au catalysts on activated carbon (Bianchi et al., 2005), graphite (Dimitratos et al., 2005a), acidic (SiO_2_, MCM-41, H-mordenite and sulphated ZrO_2_), and basic (MgO and NiO) oxide supports (Villa et al., 2015a), using the COL method. Bimetallic materials were more active than the monometallics, showing a synergistic effect between Au and Pt or Pd (Bianchi et al., 2005; Dimitratos et al., 2005a). This effect was especially significant for Pt, as it could be poisoned before full conversion. Au–Pd/C catalysts showed better selectivity to glyceric acid than Au–Pt/C. Pd mainly promoted the obtention of tartronic acid and Pt of glycolic acid. The total selectivity to glyceric acid was larger for Au–Pd/C compared to Au/C and Pd/C. At 30°C, authors obtained a very high selectivity to glyceric acid of 69% at 90% conversion with Au–Pd/C (Bianchi et al., 2005). Graphite based materials were less active (Dimitratos et al., 2005a). In terms of gold on oxides, it was found that basic MgO and NiO supports increased not only the activity, but also the reactions of C–C bond cleavage, thus decreasing the selectivity to the wanted products. However, the acidic supports led to a higher selectivity to products of C-3 oxidation. Au/MCM-41, in particular, showed a high selectivity to glyceraldehyde (Villa et al., 2015a). It is now widely accepted that the glycerol oxidation mechanism includes oxidative dehydrogenation. The β-hydride abstraction is enhanced if a base is present, this being the limiting step of the reaction (Besson and Gallezot, 2000; Mallat and Baiker, 2004; Dimitratos et al., 2012; Villa et al., 2015a).

Recently, activated carbon supported Au-Pt and Bi-Au-Pt materials were prepared by IMP by Prati's group (Motta et al., 2018). The materials were used for the oxidation of glycerol in a base free medium in mild conditions. Au-Pt/C had 68% selectivity to glyceric acid, while Bi-Au-Pt/C led to the secondary alcohol oxidation, with a selectivity of 48% to dihydroxyacetone, at 28% conversion, which is among the best values found in the literature so far.

### Oxidation of Aminoalcohols

**Scheme 4:**
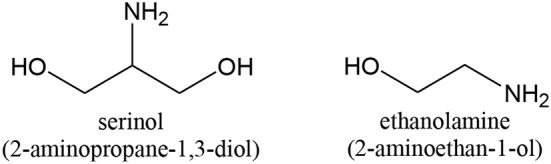
Examples of aminoalcohols used in selective oxidation reactions.

Prati's group showed that *aminoalcohols* can be transformed into aminoacids by oxidation in slightly alkaline conditions with a high selectivity (Biella et al., 2002a; Prati and Porta, 2005; Gaiassi and Prati, 2009). Au nanoparticles deposited on activated carbon (Biella et al., 2002a; Prati and Porta, 2005; Gaiassi and Prati, 2009), MgO (Gaiassi and Prati, 2009), TiO_2_ (Biella et al., 2002a; Gaiassi and Prati, 2009) and Al_2_O_3_ (Biella et al., 2002a) were used. Au was again amazingly better than other metals. The reason is that the free amino group can strongly interact with other metals, like Pd and Pt. Aminoalcohols (serinol and ethanolamine, Scheme 4) were also be oxidized to the corresponding polyols (glycerol and ethylene glycol), using Au on Al_2_O_3_, TiO_2_, MgAl_2_O_4_, and MgO (Villa et al., 2013a) by the same group.

### Oxidation of Aliphatic Alcohols

**Scheme 5:**
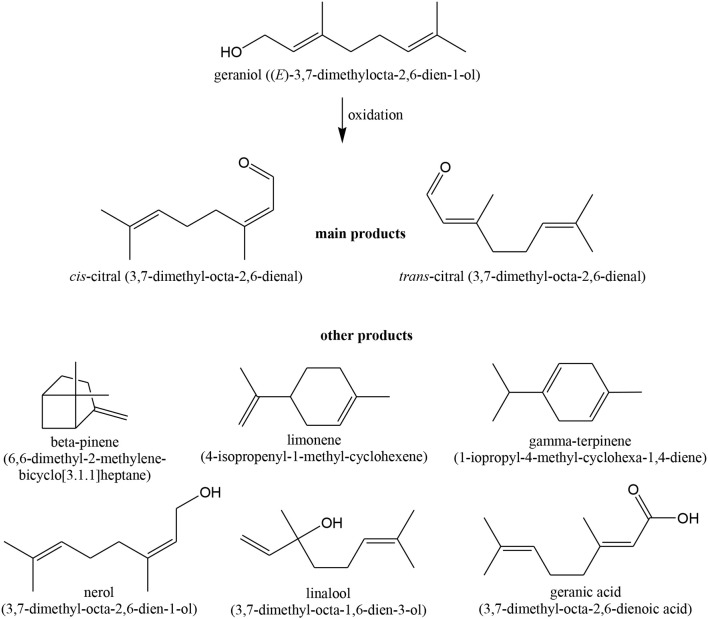
Possible products obtained in geraniol oxidation.

Hutchings and co-workers also used Au/carbon catalysts for the oxidation of *geraniol* (Scheme 5) (Hutchings et al., 2006). Cis- and trans-citral were the main products, but at higher conversions, many products such as β-pinene, limonene, γ-terpinene, linalool, nerol, and some traces of geranic acid were identified (structures seen in Scheme 5).

**Scheme 6:**
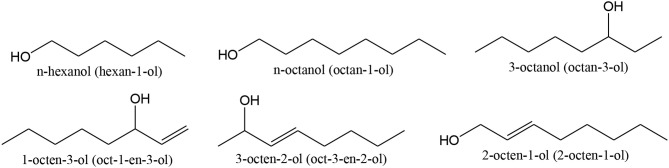
Examples of aliphatic alcohols used in selective oxidation reactions.

Corma and co-workers reported 2–5 nm sized Au nanoparticles on nanocrystalline ceria (~5 nm), as being very active, selective and recyclable for the *oxidation of several alcohols* (like *n*-hexanol, 3-octanol, 1-octen-3-ol, shown in Scheme 6), to the corresponding carbonyl products, using O_2_, atmospheric pressure, in the absence of any solvent and base (Abad et al., 2005, 2006a,b, 2008; Corma and Garcia, 2008). Au/ceria was highly selective for the *oxidation of allylic alcohols to unsaturated ketones* and was active without solvent and base (Abad et al., 2006b). Selected results can be found in Table 1. As an example, 1-octen-3-ol (Scheme 6) oxidation yielded mostly 1-octen-3-one, with a 90% selectivity, but Pd/ceria showed 58% selectivity (due to isomerization promotion and of C = C bond hydrogenation, yielding saturated ketones as a by-products) (Abad et al., 2006a).

**Table 1:** Catalytic activity of gold on ceria for the oxidation of several alcohols to the corresponding α,β-unsaturated carbonyl compounds (Abad et al., 2005; Corma and Garcia, 2008).

**Substrate**	**Conversion (%)**	**Selectivity (%)**
1-Octen-3-ol	>99	90
3-Octen-2-ol	96	91
2-Octen-1-ol	90	91
3-octanol	97	>99
n-hexanol	>99	>99

Au-Pd nanoparticles were dispersed on titania/graphene oxide (GO) composites and used for the selective oxidation of *several alcohols*, including *n*-octanol, shown in Scheme 6 (Wang et al., 2015). Similar, yet slightly better results (TOF = 228 h^−1^) were achieved for the Au-Pd/titania/GO composite, compared to the Au-Pd/titania material (TOF = 207 h^−1^).

### Oxidation of Cycloalcohols

**Scheme 7:**
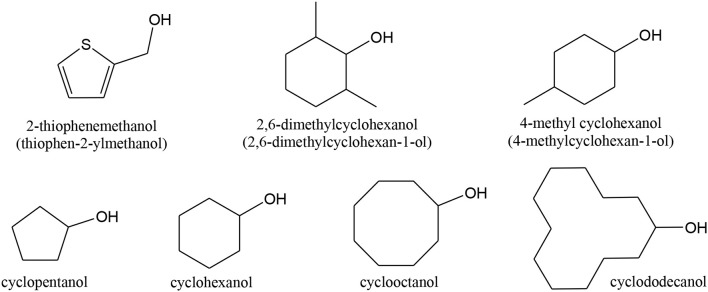
Examples of cycloalcohols used in selective oxidation reactions.

Tatsumi and co-workers (Wang H. et al., 2013) studied the selective oxidation of *cycloalcohols*, like cyclohexanol, cyclooctanol, cyclododecanol, 4-methyl cyclohexanol (Scheme 7) to aldehydes/ketones with O_2_ over Au nanoparticles on CuO, MnO_2_, NiO, CoO_x_, Fe_2_O_3_, Cr_2_O_3_, and Al_2_O_3_, TiO_2_ and SiO_2_. The catalytic activity of Au catalysts was greatly influenced by the support and the preparation method. The best results were obtained for Au/CuO co-precipitated at pH 10. The reaction might occur via oxidative dehydroxylation by direct β-CH elimination. The conversion and selectivity to ketone were above 99% for cyclooctanol and cyclododecanol. Introduction of a base greatly increased the catalyst stability. The reaction occurred via an integrated oxidation mechanism, involving the lattice oxygen of CuO.

Buonerba et al. obtained good results in the oxidation of 2-thiophenemethanol. The structures are shown in Scheme 7, using gold nanoparticles incarcerated in nanoporous syndiotactic polystyrene matrices (Buonerba et al., 2012). As syndiotactic polystyrene has a crystalline nanoporous structure, which favored the easy and selective access of the reagents to the gold catalyst located inside the polymer matrix, it was considerably accountable for the good activities found. Results suggested that the active catalysts were the ~9 nm twinned defective nanoparticles, present in large numbers.

Au-Pd nanoparticles were dispersed on titania/graphene oxide composites and used for the selective oxidation of cyclohexanol (Scheme 7) (Wang et al., 2015). Better results (TOF = 4,700 h^−1^) were achieved for the Au-Pd/titania/GO composite, compared to the Au-Pd/titania material (TOF = 4,130 h^−1^).

Corma and co-workers reported 2–5 nm sized Au nanoparticles on nanocrystalline ceria (~5 nm), as being very active, selective, and recyclable for the *oxidation of* 2,6-dimethylcyclohexanol (Scheme 7), using O_2_, atmospheric pressure, in the absence of any solvent and base (Abad et al., 2005). 78% conversion with 94% selectivity to 2,6-dimethylcyclohexanone was achieved in 2.5 h reaction time.

### Oxidation of Aromatic Alcohols

**Scheme 8:**
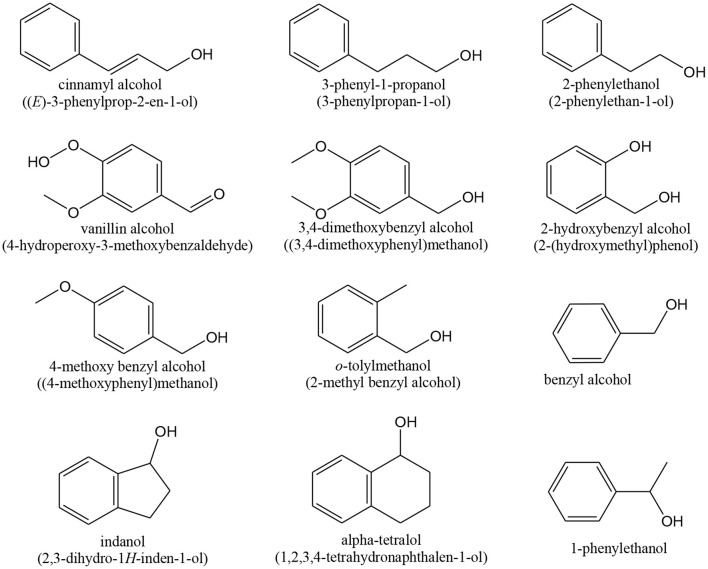
Examples of aromatic alcohols used in selective oxidation reactions.

The first report on the use of a gold catalyst for alcohol oxidation dates from 1992 and deals with the oxidation of an aromatic alcohol (4-methoxybenzyl alcohol, shown in Scheme 8) to the corresponding aldehyde, performed by a [Au(IO_5_(OH))_2_]^5−^ complex (Dengel et al., 1992).

Corma and co-workers reported 2–5 nm sized Au nanoparticles on nanocrystalline ceria (~5 nm), as being very active, selective and recyclable for the *oxidation of several alcohols* (like 2-phenylethanol, cinnamyl alcohol, 3,4-dimethoxybenzyl alcohol, 3-phenyl-1-propanol, vanillin alcohol, 2-hydroxybenzyl alcohol, shown in Scheme 8), using O_2_, atmospheric pressure, in the absence of any solvent and base (Abad et al., 2005, 2006a,b, 2008; Corma and Garcia, 2008). Selected results are shown in Table 2. Milder conditions are needed and better results are obtained for the oxidation of cinnamyl and 3,4-dimethoxybenzyl alcohols to the corresponding acids, than to the corresponding aldehydes.

**Table 2:** Catalytic activity of gold on ceria for the oxidation of several alcohols to the corresponding carbonyl compounds (Abad et al., 2005).

**Substrate (S)**	**Time (h)**	**Conversion (%)**	**Product**	**Selectivity (%)**
2-phenylethanol^a^	2.5	92	acetophenone	97
cinnamyl alcohol^a^	7	66	cinnamaldehyde	73
cinnamyl alcohol^b^	3	>99	cinnamylic acid	98
3,4-dimethoxybenzyl alcohol^a^	7	73	3,4-dimethoxybenzaldehyde	83
3,4-dimethoxybenzyl alcohol^b^	2	>99	3,4-dimethoxybenzylic acid	>99
3-phenyl-1-propanol^a^	6	70	3-phenylpropyl 3-phenylpropanoate	98
vanillin alcohol^b^	2	96	vanillin	98
2-hydroxybenzyl alcohol^b^	2	>99	2-hydroxybenzaldehyde	87

a *Substrate (4.85 mmol), Au/CeO_2_ (0.5 mol %), 353 K, p = 1 atm O_2_ (flow: 25 mL min^−1^)*.

b *Substrate (0.4 mmol), Au/CeO_2_ (0.66 mol %), H_2_O (5 mL), Na_2_CO_3_ (0.3 g), 323 K, p = 1 atm O_2_ (flow: 25 mL m^−1^)*.

Wu X. C. et al. (2016) used nanocomposites of graphene quantum dots and Au nanoparticles immobilized on Fe_3_O_4_ nanoparticles (GQDs/Au/Fe_3_O_4_ ternary composites with superparamagnetic properties being easy to remove from the reaction mixture) for the solvent-free *oxidation of aromatic alcohols* (containing an aromatic benzyl group) with air as oxidant. Materials showed good catalytic performance with the aromatic alcohols being oxidized to the corresponding aldehydes with high selectivity (>99%) and conversion.

Au-Pd nanoparticles were dispersed on titania/graphene oxide composites and used for the selective oxidation of benzyl alcohol, 4-methoxy benzyl alcohol, cinnamyl alcohol, 1-phenylethanol, shown in Scheme 8 (Wang et al., 2015). Results are shown in Table 3. The resulting optimized catalyst showed activities compared to the Au-Pd/TiO_2_ material prepared by COL (although much better for 4-methoxy benzyl alcohol), but the GO composite was more stable and could reused for three cycles without loss of activity.

**Table 3:** Alcohol oxidation for supported Au-Pd Catalysts (Wang et al., 2015).

**Substrate**	**Catalyst**	**TOF (h^**−1**^)**
benzyl alcohol	Au-Pd/TiO_2_ Au-Pd/TiO_2_/GO	10,300 10,400
4-methoxy benzyl alcohol	Au-Pd/TiO_2_ Au-Pd/TiO_2_/GO	12,800 15,000
cinnamyl alcohol	Au-Pd/TiO_2_ Au-Pd/TiO_2_/GO	3,360 4,070
1-phenylethanol	Au-Pd/TiO_2_ Au-Pd/TiO_2_/GO	6,000 6,390

Tatsumi and co-workers (Wang H. et al., 2013) studied the selective oxidation of *cycloalcohols*, like benzyl alcohol, 2-methyl benzyl alcohol, 4-methyl benzyl alcohol, cinnamyl alcohol (Scheme 8) to aldehydes/ketones with O_2_ over Au nanoparticles on CuO, MnO_2_, NiO, CoO_x_, Fe_2_O_3_, Cr_2_O_3_, and Al_2_O_3_, TiO_2_ and SiO_2_. A larger amount of methyl groups lead to an activity increase. The catalytic of Au catalysts was greatly influenced by the support and the preparation method. The best results were obtained for Au/CuO co-precipitated at pH 10. The reaction might occur via oxidative dehydroxylation by direct β-CH elimination. Giorgi et al. also showed that Au/alumina could also be efficiently used in the oxidation of benzylic (and allylic alcohols) under O_2_, in good yields (68–99%) and with excellent selectivity (ca. 100%) (Giorgi et al., 2017).

Buonerba et al. obtained good results in the oxidation of cinnamyl alcohol, indanol, and α-tetralol (secondary alcohols). The structures are shown in Scheme 8, using gold nanoparticles incarcerated in nanoporous syndiotactic polystyrene matrices (Buonerba et al., 2012). As said above, the crystalline nanoporous structure of syndiotactic polystyrene favored access of the reagents to the gold catalyst located inside the polymer matrix, improving activity. The active catalysts were the abundant ~9 nm twinned defective nanoparticles.

**Scheme 9:**
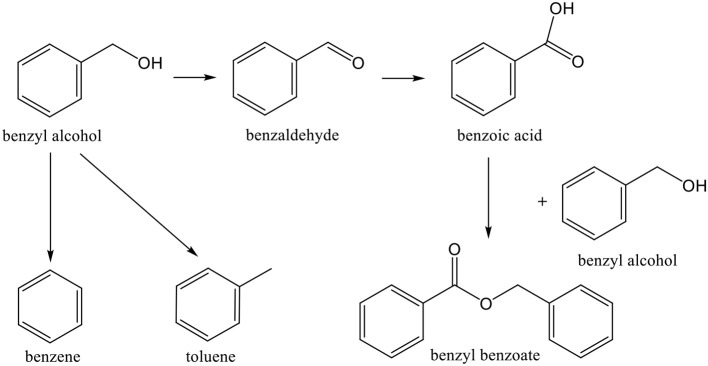
Reactions of benzyl alcohol oxidation.

Miyamura et al. also proved the catalytic activity of polymer-supported gold for “greener” liquid phase selective *oxidation of several aromatic alcohols*, like phenylmethanol (benzyl alcohol) and 1-phenylethanol (Scheme 9) and cyclopentanol (Scheme 7) (Miyamura et al., 2007). Those materials showed higher activity than Au on metal oxides. Choudhary and Dumbre also tested similar *aromatic alcohols*, using a Au/MgO catalyst prepared by DP (Choudhary and Dumbre, 2011). The highest activity was found for the oxidation of 4-methoxy benzyl alcohol (Scheme 8) with 68% conversion (and a 95% selectivity to the aldehyde). Fristrup and co-workers discussed the substituted benzyl alcohols aerobic oxidation mechanism and concluded that the rate-determining step involved hydride abstraction, that is, the formation of a partial positive charge in the benzylic moiety (Fristrup et al., 2008).

**Scheme 10:**
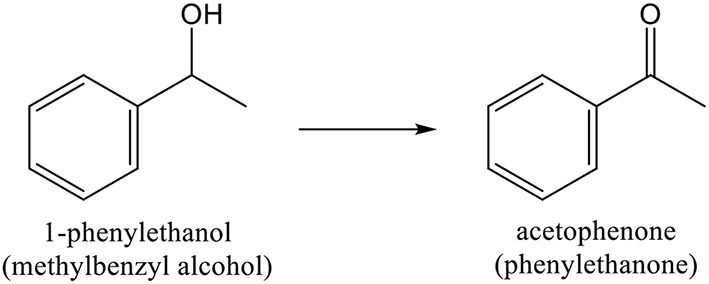
Oxidation of 1-phenylethanol to acetophenone.

Among aromatic alcohols, the already referred *benzyl alcohol* (Scheme 9) and *methylbenzyl alcohol* (Scheme 10) are low toxic naturally produced examples. Their partial oxidation can yield *benzaldehyde* (Scheme 9) and *acetophenone* (Scheme 10), respectively. These products have a large importance in industrial organic synthesis (since they are precursors to other organic compounds, ranging from plastic additives to pharmaceuticals).

Gold catalysts have been successfully used for oxidation of *benzyl alcohol* to benzaldehyde (Choudhary et al., 2005, 2007, 2009; Hutchings et al., 2006; Su et al., 2007; Mitsudome et al., 2009; Zhu et al., 2010; Prati et al., 2011; Xie et al., 2012; Xu et al., 2012; He et al., 2013; Wang H. et al., 2013; Yu et al., 2013; Alhumaimess et al., 2014; Hong et al., 2014; Morad et al., 2014; Movahed et al., 2014; Silva T. A. G. et al., 2014; Nepak and Srinivas, 2015; Ferraz et al., 2016; Sun et al., 2016; Giorgi et al., 2017; Liu et al., 2018; Gualteros et al., 2019). Other formed by-products can be toluene, benzene and benzoic acid (Prati et al., 2011; Wang et al., 2015), as seen in Scheme 8. Choudhary et al. (Choudhary et al., 2005, 2007, 2009; Choudhary and Dumbre, 2009a,b, 2010, 2011) were one of the first groups to study this oxidation reaction. They used Au on MgO, BaO, CaO, and SrO (alkaline earth oxides), Al_2_O_3_, In_2_O_3_, Ga_2_O_3_, and Tl_2_O_3_ (group IIIa metal oxides), TiO_2_, U_3_O_8_, Cr_2_O_3_, Fe_2_O_3_, CoO_x_, MnO_2_, CuO, ZnO, NiO, Y_2_O_3_, and ZrO_2_ (transition metal oxides), Ce_2_O_3_, La_2_O_3_, Sm_2_O_3_, Eu_2_O_3_, Tb_2_O_3_, Er_2_O_3_ Nd_2_O_3_, and Yb_2_O_3_ (rare earth metal oxides) prepared by DP, for the liquid-phase oxidation of benzyl alcohol to benzaldehyde. The Au/TiO_2_ and Au/ZrO_2_ catalysts showed high activity and selectivity for the reaction.

Other authors used Au on CuO (Wang H. et al., 2013), MnO_2_ (Alhumaimess et al., 2014), TiO_2_ (Ferraz et al., 2016), and titanate nanotubes (Nepak and Srinivas, 2015). Su et al. found that gold on Ga_3_Al_3_O_9_ can be very efficient and active for the benzyl alcohol oxidation at room temperature (Su et al., 2007). Carabineiro and co-authors used gold on different metal oxide supports by DP (Al_2_O_3_, Fe_2_O_3_, ZnO, and TiO_2_) (Martins et al., 2017). The obtained materials were tested for the benzyl alcohol oxidation using *tert*-butyl hydroperoxide (TBHP) as oxidant, for 1 h, under microwave irradiation, at 100°C. The materials exhibited good activity for benzaldehyde formation, with no traces of by-products. The use of microwave is regarded as much more effective, when compared with conventional heating, usually with similar yields achieved in a shorter time and at lower temperatures (Varma, 2007; Dudley et al., 2015). Other authors also used TBHP (Choudhary and Dumbre, 2009a,b, 2010; Choudhary et al., 2009; Li H. R. et al., 2009; Peneau et al., 2013; Zhang B. et al., 2016; Martins et al., 2017; Ndolomingo and Meijboom, 2017; Gogoi et al., 2018; Kashani et al., 2018) and H_2_O_2_ (Zhan et al., 2012, 2013; Hallett-Tapley et al., 2013; Moreno et al., 2013; Santonastaso et al., 2014; Tang et al., 2014; Long and Quan, 2015; Mehri et al., 2015; Restrepo et al., 2015a,b; Emayavaramban et al., 2016a; Zhang B. et al., 2016; Gogoi et al., 2018; Khawaji and Chadwick, 2018; Tareq et al., 2018) as oxidants for alcohol oxidation reactions, although more studies deal with the use of oxygen (Besson and Gallezot, 2000; Prati and Porta, 2005; Bond et al., 2006; Hashmi and Hutchings, 2006; Hutchings et al., 2006; Carabineiro and Thompson, 2007, 2010; Ishida and Haruta, 2007; Fristrup et al., 2008; Della Pina and Falletta, 2011; Della Pina et al., 2012; Dimitratos et al., 2012; Takei et al., 2012; Hutchings, 2014; Freakley et al., 2015; Sharma et al., 2016).

Recently, gold nanoparticles on alumina, silica and titration, prepared by DP with urea, for the oxidation of benzyl alcohol, in the absence of solvent, with low metal (0.08–0.05 mol% of Au) loadings, using O_2_ as oxidant (Gualteros et al., 2019). A small amount of base was enough to activate the catalyst. Au/Al_2_O_3_ showed a good catalytic performance (TOF = 443,624 h^−1^ at 100°C) for 0.08 mol% Au loading, in optimized conditions, being the most stable material, being stable up to 5 cycles.

Carbon materials have also been utilized. Hutchings and co-workers used Au/activated, which showed high selectivity at low conversion (Hutchings et al., 2006). Benzyl alcohol oxidation was also studied on Au/CNT and Au/CNF by Prati's group (Prati et al., 2011). Gold on carbon xerogels (Xu et al., 2012) and on graphene derivatives (Xie et al., 2012; Yu et al., 2013; Movahed et al., 2014) has also been reported. Carabineiro and co-authors also tested the liquid phase selective oxidation of benzyl alcohol on Au/activated carbon and Au/C_3_N_4_ (Zhu et al., 2010). The catalyst without oxygen showed negligible activity for oxidation reactions, showing that the metal only is ineffective for the activation of molecular oxygen. Also it was found that the oxidation activity depended on the amount of oxygen containing species of the catalyst, suggesting that the oxygen sites are where molecular oxygen adsorption and activation take place.

Bimetallic Au-Pd (Hong et al., 2014; Morad et al., 2014; Silva T. A. G. et al., 2014; Sun et al., 2016) and trimetallic Au-Pd-Pt (He et al., 2013) catalysts have also been reported by several authors. They showed significant enhanced activity, compared to monometallic Au and Pd materials. The addition of Pt promoted the selectivity to benzaldehyde, suppressing toluene formation (He et al., 2013).

The oxidation of *1-phenylethanol* (methylbenzyl alcohol) to acetophenone (phenylthenone), shown in Scheme 10, has also been studied on gold catalysts (Abad et al., 2005; Miyamura et al., 2007; Haider et al., 2009; Mitsudome et al., 2009; Ni et al., 2009; Wang et al., 2010; Buonerba et al., 2012; Hosseini-Monfared et al., 2013; Imura et al., 2016; Wang S. et al., 2016; Martins et al., 2017). For example, Corma and co-workers reported that Au/ceria catalysts showed a TOF value of 12,480 h^−1^, at 160 °C, with >99 % selectivity, for this reaction (Abad et al., 2005). That value was larger than the reported for Pd on hydroxyapatite (9,800 h^−1^), as reported by Mori et al. (2004).

Takato and co-workers (Mitsudome et al., 2009) observed that a hydrotalcite supported nanoparticle (Au/HT) was a good heterogeneous catalyst for the oxidation of 1-phenylethanol under mild conditions. The turnover number (TON, mol of product per mol of Au catalyst) and TOF were 200,000 and 8,300 h^−1^, respectively. Moreover, the catalyst could be effortlessly filtrated and recycled without much loss of activity and selectivity. Imura et al. used surface clean Au nanoflowers and Au nanoparticles supported on γ-Al_2_O_3_ (Imura et al., 2016). The formation rate of acetophenone on nanoflowers was 10-fold higher than on (spherical) nanoparticles with a similar diameter.

As referred above, Buonerba et al. used gold nanoparticles incarcerated in nanoporous syndiotactic polystyrene matrices to study the oxidation of several alcohols, suggesting that nanoparticles of ~9 nm diameter were the active catalyst (Buonerba et al., 2012). High yields (96%) of acetophenone were obtained in 1 h, at 35°C. Haider et al. used Au nanoparticles on CuMg_2_Al_1_O_x_ and also found higher activity for gold particles of *ca*. 9 nm, with yields near 90%, at 90°C for 1 h (Haider et al., 2009).

Hosseini-Monfared et al. used gold nanoparticles with a similar size (8 nm average) dispersed in 1-n-butyl-3-methylimidazolium tetrafluoroborate ionic liquid and found 100% selectivity to acetophenone, with intermediate α-hydroxy carbon radical formation (Hosseini-Monfared et al., 2013). The TON was 200. This value could be increased with the addition of N-hydroxyphthalimide, but at a cost of a selectivity drop to 58%. Tests were carried out at 100°C, under 4 bar of O_2_, without any base. However, the use of molecular oxygen should be undertaken with proper safety precautions, as reported by other authors (Bay et al., 2016).

Wang et al. used gold nanoparticles supported on a layered double hydroxide and obtained a acetophenone around 100%, using O_2_, atmospheric pressure, room temperature, 2 h conditions (Wang et al., 2010). Upon 6 recycling cycles, the activity dropped to 97%. The gold nanoparticles had sizes in the 1–5 nm range. Furthermore, Ni et al. reported an efficient H_2_O_2_-Au approach for the 1-phenylethanol oxidation under solvent free conditions, which was considered as a “green” oxidation of heterogeneous metal complexes (Ni et al., 2009). For example, the Au/TiO_2_ catalyst obtained a conversion of 99%, and the yield of acetophenone was between 98 and 100%, which means that the formation of well-dispersed Au nanoparticles, together with a beneficial interaction with the TiO_2_ support, is the major factor for obtaining high activity in the H_2_O_2_ mediated oxidation of 1-phenylethanol.

Nickel-containing layered double hydroxides supporting atomic precise Au-25 nanoclusters were reported by other authors (Wang S. et al., 2016). The catalysts exhibited excellent activity for selective oxidation of 1-phenylethanol to acetophenone, with O_2_, under base-free conditions. The highest activity showed a TOF of 118,500 h^−1^ in a solvent-free environment and could be applied for a wide range of alcohols. The material could be recycled 5 times without mich loss of activity.

**Figure 4:**
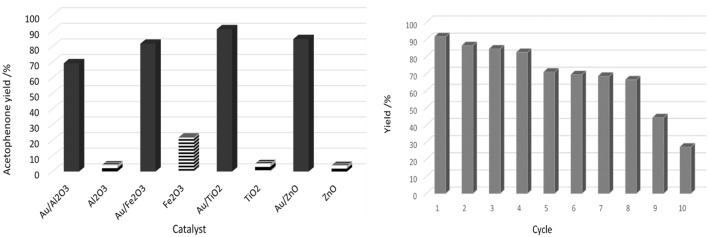
**(Left)** Comparison of the acetophenone yield obtained by microwave assisted 1-phenylethanol oxidation with TBHP, using Au nanoparticles supported at different oxides and the metal oxides as catalysts. Conditions: 100°C, 1 h, 10 W microwave, 600 rpm. **(Right)** Recyclability of the Au/TiO_2_ affecting the yield of acetophenone for the microwave assisted oxidation of 1-phenylethanol. Conditions: 100°C, 1 h, 10 W microwave, 600 rpm. Copyright (2017) Wiley. Adapted with permission from Martins et al. (2017).

Carabineiro and co-authors used gold loaded on different metal oxide supports (Al_2_O_3_, Fe_2_O_3_, ZnO, and TiO_2_), by DP, in the oxidation of 1-phenylethanol, using TBHP as oxidant, under microwave irradiation (Martins et al., 2017). Those catalytic systems exhibited good activity in the formation of acetophenone (Figure 4, left). No traces of by-products were found. Adding Au increased the alcohol conversion from 5% (TiO_2_) to 91% (Au/TiO_2_), which was the best result obtained in this study. Au/TiO_2_ recyclability was tested up to a maximum of 10 cycles and the catalytic activity was very high in the initial 4 cycles (Figure 4, right). The loss of activity was due to a large increase in gold nanoparticle size and gold leaching (in the 10th cycle).

## Alkane Oxidation

Hydrocarbons, in particular alkanes, are the main components of gas and oil. The C-H bond(s) of these compounds can be transformed into C-OH or C = O groups that will lead to the production of high added value products that will have applications in fine chemistry. The selective oxidation of hydrocarbons is a very important reaction taking place in industrial processes based on petroleum, since the oxygenated compounds produced can be used as intermediates for organic synthesis (Kalvachev et al., 1999). However, it is difficult to activate such bonds in these very stable compounds, and that prevents that they are more commonly used in the synthesis of other important products (Weissermel and Arpe, 1993; Derouane et al., 1998, 2005; Clark and Macquarrie, 2002; Sheldon et al., 2007).

### Oxidation of Cyclohexane

**Scheme 11:**
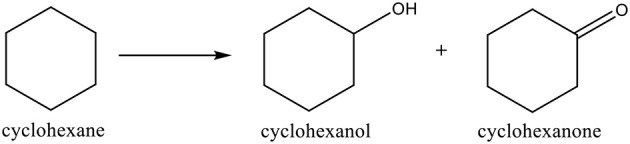
Oxidation of cyclohexane to cyclohexanol and cyclohexanone.

A good example with increasing industrial importance is the *oxidation of cyclohexane to cyclohexanol and cyclohexanone* (Scheme 11), that are important compounds to be used in the production of caprolactam and adipic acid, utilized in the nylon-6 and nylon-66 polymers manufacture. These products can also be used as solvents, homogenizers, and stabilizers (Carabineiro and Thompson, 2007). The cyclohexanol and cyclohexanone mixture is also called KA (ketone-alcohol) oil. The industrial process for KA oil production includes a homogeneous cobalt catalyst and O_2_ as oxidant, at high temperature (150°C), with products being formed at low yields (4–12%), with only 80–85% selectivity (Weissermel and Arpe, 1993; Whyman, 2001; Clark and Macquarrie, 2002; Mears and Eastman, 2004; Derouane et al., 2005). Thus, there is a need for more effective systems to be used under milder conditions (Weissermel and Arpe, 1993; Schuchardt et al., 2001; Clark and Macquarrie, 2002; Shulpin, 2009; Kirillov and Shul'pin, 2013).

Shulpin first studied the (photo)oxidation of cyclohexane with oxygen (Lederer et al., 1992; Nizova and Shulpin, 1992; Shulpin and Nizova, 1993) and oxidation of other alkanes with H_2_O_2_ (Shul'pin et al., 2001), using gold chloride compounds. Not much studies on cyclohexane oxidation have been done afterwards with gold complexes, apart from the work of Carabineiro and co-authors (Peixoto De Almeida et al., 2013; Carabineiro et al., 2018). Moreover, the oxidation of other alkanes (with gold complexes) is also scarce (Nikitenko and Shestakov, 2013).

The first reports dealing with the selective oxidation of cyclohexane to cyclohexanone and cyclohexanol using Au supported catalysts were reported by Suo and co-workers in 2004 (Lu et al., 2004, 2005; Zhao et al., 2004). The initial studies were carried out using a calcined Au/ZSM-5 catalyst, with O_2_, without solvent (Zhao et al., 2004). Authors reported that this catalyst was very active and could be used up to two cycles without much loss of activity. The yield decreased with temperature (from 140 to 180°C), and also the total selectivity of cyclohexanol and cyclohexanone (at 180°C) (Zhao et al., 2004). Those authors studied this reaction over a Au/MCM-41 catalyst, also in the absence of solvent, with 1 MPa O_2_, at 140–160°C, for 6–8 h (Lu et al., 2004, 2005). The conversion was ~16% and the selectivity to cyclohexanone up to ~76%. Authors claimed that their work was the first reporting such excellent values of conversion and selectivity for these reaction systems. The catalyst could be recycled for at least three times, without much loss of conversion and selectivity (Lu et al., 2004, 2005).

In 2005; Hutchings et al. showed that Au/graphite could also be used to promote this reaction, using TBHP as initiator (Xu et al., 2005; Hutchings et al., 2006). A modest activity at 70°C and 0.3 MPa O_2_ was reported, with high selectivities to cyclohexanol and cyclohexanone only for very low conversions, after 17 h of reaction (Xu et al., 2005). The work of those authors was the only reference to Au/carbon combination for cyclohexane oxidation for some time.

**Figure 5:**
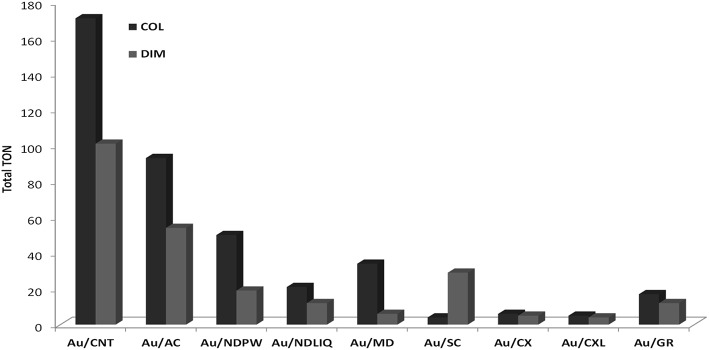
Dependence of the overall TON (moles of cyclohexanol + cyclohexanone per mole of Au nanoparticles loaded on the carbon material) of the products on the type of support and impregnation method. Reaction conditions: CH_3_CN (3.0 mL), cyclohexane (5.0 mmol), n_pyrazinecarboxylicacid_/n_catalyst_ (50), room temperature, 6 h. Reprinted from Carabineiro et al. (2013). Copyright (2013), with permission from Elsevier.

In 2013; Carabineiro et al. tested gold nanoparticles on several *carbon supports*: activated carbon (AC), carbon xerogels—two different samples, one with smaller mesopore width (13.6 nm), prepared at pH = 6 (CX), and another with larger width (32.3 nm), prepared at pH = 5.5 (CXL), carbon nanotubes (CNT), microdiamonds (MD) and nanodiamonds (ND) in powder (NDPW) and liquid dispersion (NDLIQ), graphite (GR), and silicon carbide (SC) (Carabineiro et al., 2013). Gold was loaded by DIM and COL. The materials were tested at room temperature and atmospheric pressure, using a “green” oxidant (H_2_O_2_). Au/CNT-COL was the most active catalyst (Figure 5), with a yield of 3.6% and a TON of ~171 (6 h reaction). The yield is similar to the industrial process (which needs high amounts of a Co catalyst and 150°C), but has the advantage of being obtained at room temperature and much lower amount of catalyst (Au catalyst/substrate molar ratio below 1 x 10^−3^), being thus “greener.” Also very high selectivity toward the formation of cyclohexanol and cyclohexanone was obtained, without any by-products. The 3.6% yield obtained for this sample is similar to that reported by Hutchings and co-workers (3.7%) for 1% Au on graphite (Xu et al., 2005). But these authors needed 0.3 MPa O_2_, 17 h reaction and 70°C, and the total selectivity for KA oil was 23.1%. Only for low conversions (~1%), higher selectivities (~91.6%) could be obtained, under the same conditions, with with 0.5% Au and with TBHP as additive (Xu et al., 2005). Therefore, the results of Carabineiro et al. (2013), showing a higher selectivity and a similar yield are more favorable, environmentally friendly and adequate for industry.

It was shown that an acidic medium could have a promoting effect as also found in previous studies dealing with homogeneous (Nizova et al., 2002; Shul'pin et al., 2004; Alegria et al., 2007; Silva et al., 2008, 2010, 2011; Fernandes et al., 2009; Mishra et al., 2009), and supported complexes (Mishra et al., 2008) and other metal catalysts (Kirillov and Shul'pin, 2013). The used pyrazine carboxylic acid might activate the metal center by protonation of a ligand, causing further unsaturation, enhance the oxidation capacity of metal complexes, and stabilize the peroxide preventing decomposition and promoting the formation of peroxo (or hydroperoxo)-complexes (Carabineiro et al., 2018). The recycling tests showed that the best catalyst was able to maintain the high activity up to five cycles, with very high selectivity and no leaching.

Liu et al. used Au nanoparticles on CNT composites for the photocatalytic oxidation of cyclohexane, achieving ~14.6% conversion with a selectivity of ~86.9% to cyclohexanol, using air and visible light, at room temperature (Liu et al., 2014a). Other authors also used Au nanoparticles on carbon quantum dots as photocatalysts, achieving a conversion of 63.8% and a selectivity of 99.9% to cyclohexane and cyclohexanone, using H_2_O_2_, at room temperature, under visible light (Liu R. H. et al., 2014). Kang and co-workers also tested the photocatalytic oxidation of cyclohexane using Au on carbon nitride (C_3_N_4_) and obtained 10.54% conversion and 100% selectivity to cyclohexanone without the need of initiator or oxidant, under visible light (Liu et al., 2014b). Authors showed that C_3_N_4_ could photocatalyse water oxidation to generate H_2_O_2_, which would then act as oxidant.

More recently, Mayani et al. reported on Au, Pd and and Au-Pd anchored carbon composites with 25 and 170 nm size carbon cages, synthesized using nano-silica sphere templates and fuel oil from pyrolysis of pitch residue as carbon source (Mayani et al., 2016). Such materials were used for cyclohexane oxidation at room temperature and atmospheric pressure, using H_2_O_2_ as oxidant, in N_2_ atmosphere. The most active catalyst (Au-based) showed a yield of 7.7% after 4 h reaction, superior to Pd- and Au-Pd- based analogs. Recyclability did not show much activity loss.

*Metal oxides* have also been referred as supports for Au for the same reaction (Zhu et al., 2005; Xu et al., 2007a,b, 2008; Carneiro et al., 2009, 2011; Li et al., 2009a,b; Xie et al., 2009, 2011; Hereijgers and Weckhuysen, 2010; Wu P. P. et al., 2010; Wan et al., 2011; Wu et al., 2011a,b,c, 2016b; Alshammari et al., 2012, 2015, 2016; Conte et al., 2012; Sun et al., 2012; Sannino et al., 2013; Wang C. H. et al., 2013; Zhou et al., 2014; Gui et al., 2015; Liu et al., 2015; Mohamed, 2015; Chen L. F. et al., 2017; Martins et al., 2017). Most studies were carried out using O_2_ as oxidant (Zhu et al., 2005; Xu et al., 2007a,b, 2008; Li et al., 2009a; Xie et al., 2009, 2011; Hereijgers and Weckhuysen, 2010; Wu P. P. et al., 2010; Wan et al., 2011; Wu et al., 2011a,b,c, 2016b; Alshammari et al., 2012; Sun et al., 2012; Wang C. H. et al., 2013; Zhou et al., 2014; Gui et al., 2015; Chen L. F. et al., 2017). Many reports refer the use of TiO_2_ based materials (Carneiro et al., 2009, 2011; Hereijgers and Weckhuysen, 2010; Alshammari et al., 2012; Sun et al., 2012; Sannino et al., 2013; Zhou et al., 2014; Martins et al., 2017). Interestingly, for the photo-oxidation of cyclohexane with air, the photocatalytic activity of TiO_2_ (Hombikat) was not enhanced by Au deposition, as shown by Mul and co-workers (Carneiro et al., 2009, 2011). The reason is that the deposition of gold caused a large decrease in the amount of OH- groups of the support, suggesting that such moieties were more determinant for the catalytic activity than the presence or absence of Au (Carneiro et al., 2009).

However, the photocatalytic cyclohexane partial oxidation in the gas-phase was effectively achieved on Au/TiO_2_ (Sannino et al., 2013). The products obtained were cyclohexanol, cyclohexanone, and CO_2_. Authors showed that an increase in the Au content, could revert the process selectivity from cyclohexanol (75%) to cyclohexanone (80%).

Mohamed also reported on the photocatalytic oxidation of cyclohexane, with H_2_O_2_ as oxidant, using gold on reduced graphene oxide (Au/rGO), titania nanotubes (Au/TNT) and titania nanotubes-multi-walled carbon nanotubes composites (Au/TNT–CNT), under UV irradiation (Mohamed, 2015). Both Au/rGO and Au/TNT–CNT can promote the oxidation with conversions ranging from 6 to 9.0% and selectivities from 60 to 75% for cyclohexanone, with the latter giving the best result. The oxidation followed a radical-chain mechanism.

Silica-based materials have also been used (Zhao et al., 2005; Zhu et al., 2005; Xu et al., 2007b, 2008; Li et al., 2009a; Wu et al., 2011a,b,c, 2016b; Wang C. H. et al., 2013; Zhou et al., 2014; Gui et al., 2015; Saxena et al., 2016; Chen L. F. et al., 2017). Au/mesoporous silica showed high catalytic activity and selectivity for cyclohexane oxidation using O_2_ in solvent-free conditions (Zhu et al., 2005; Wu et al., 2011a,b,c, 2016b). Li et al. used Au nanoparticles (3–8 nm) on SBA-15, under an O_2_/N_2_ atmosphere (Li et al., 2009b). At 1.0 MPa and 150°C, when the reaction time increased from 3 to 6 h, conversion also rose from 15 to 20%, but with an overall decline of selectivity to KA-oil.

Cyclohexane oxidation was also tested over a Au/SiO_2_ catalyst, with propylene carbonate significantly enhancing the reaction of cyclohexane oxidation to 21.9% conversion, while maintaining a high selectivity of 83.2% toward KA oil, using O_2_ as oxidant and TBHP as initiator, at 140°C, for 2 h (Gui et al., 2015). The effect of propylene carbonate can be attributed to its high polarity and it can facilitate the reaction by promoting the decomposition of cyclohexyl hydroperoxide. Recycling tests showed no significant changes in the conversion of cyclohexane and selectivity to KA oil up to four reaction cycles, with no Au leaching. Gold nanoparticles on amorphous silica were used and gave a 22.7% conversion and 80.6% selectivity to cyclohexanol and cyclohexanone, under dipolar non-hydrogen bond donor acetone solvent, at 150°C, 1.5 MPa O_2_, after 3 h (Wang C. H. et al., 2013).

Silica-titania supported gold catalysts were tested at 150°C, 1.5 MPa O_2_ and 3 h reaction, achieving a 91.7% selectivity with a conversion of 8.4% (Xu et al., 2007b). In similar conditions, Au on TiO_2_/MCM-41 was promising for the cyclohexane oxidation, achieving a TOF of 29,145 h^−1^ with ~9.9% conversion of cyclohexane (Zhou et al., 2014). Gold nanoparticles on silica-alumina were also used, in the absence of any solvent and initiator, achieving a 9.8% conversion and a 88.8% selectivity to KA oil, at 150 °C, 1.5 MPa O_2_, after 3 h (Xu et al., 2008).

Gold nanoparticles (<2 nm diameter) were highly dispersed and well-confined in the hybrid shells of silica nanospheres, through the anchorage of organic functional groups, under a condensation process (Chen L. F. et al., 2017). The materials exhibited good catalytic activity for solvent-free catalytic oxidation of cyclohexane with 94.8% selectivity to KA oil and adipic acid, at 150°C, 1.5 MPa O_2_, after 3 h.

Saxena et al. encapsulated gold nanoparticles by silica, further encapsulated them with zeolites (MCM-22 and ZSM-5) nanoshells and used the resulting material for the oxidation of cyclohexane, at 150°C, under 1 MPa O_2_ pressure, in a solvent-free system, for 2 h (Saxena et al., 2016). Au@MCM-22 exhibited the highest TON (1788) and TOF (596 h^−1^) among the analyzed samples, due to a higher concentration of strong acid sites. The nano-capsules acted as bifunctional catalysts, with the nanoparticles prevented from agglomeration during synthesis or catalytic applications, and the zeolitic-shell enhanced conversion and reusability of the nanocatalysts.

Alshammari et al. compared Au/CaO, Au/MgO, Au/ZrO_2_, Au/TiO_2_, Au/Al_2_O_3_ (Alshammari et al., 2012, 2016). A conversion of over 25 %, with 70% selectivity to KA oil, was achieved over a Au/TiO_2_ (anatase) catalyst, in the temperature range of 100°C, at 10 bar O_2_, with TBHP as initiator. The high activity of this material was due to the smaller size (2 nm) of Au nanoparticles.

9.07% conversion of cyclohexane and 91.90% selectivity to KA oil were obtained using 3.0% /Co_3_O_4_ as catalyst, at 150°C, after 3 h, with 1.5 MPa O_2_ (Wan et al., 2011). Under similar conditions, 0.2% Au/Al_2_O_3_ achieved 12.6% conversion, with 84.7% selectivity to the ketone and alcohol mixture (Xu et al., 2007a).

Au nanoparticles supported on Cr-based metal-organic frameworks (MOFs), and other oxides, like TiO_2_ and Fe_2_O_3_, prepared by DP with urea, were used for the reaction using O_2_, without solvent and initiator (Sun et al., 2012). The best result was 30.5% conversion, 26.7% yield, with 87.7% selectivity to KA oil, for Au on a Cr-MIL-101 type of MOF.

**Figure 6:**
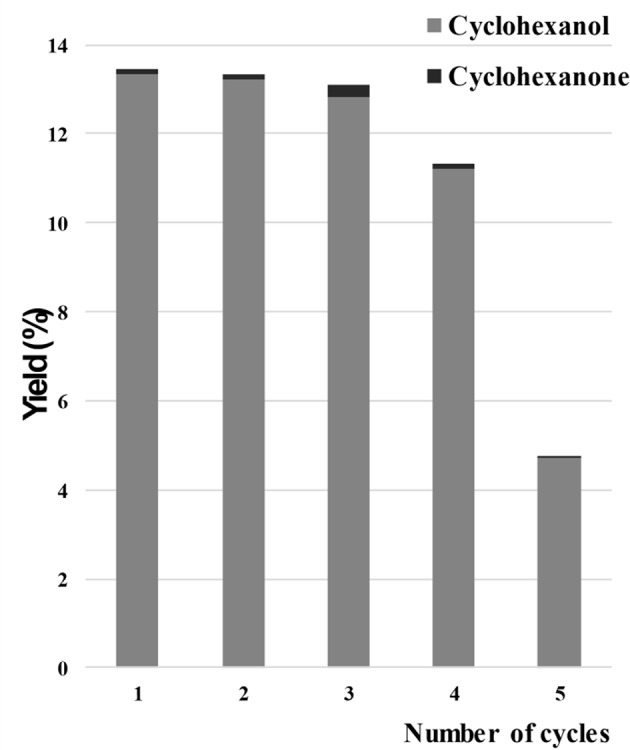
Effect of the Au/Fe_2_O_3_ recycling on the yield of cyclohexanol and cyclohexanone for the cyclohexane oxidation. Reaction conditions: cyclohexane (0.25 M), catalyst (10 mmol of Au on iron oxide, 0.4 mol% vs. substrate), H_2_O_2_ (1.2 M), in acetonitrile, at 60°C, for 4 h. Copyright (2017) Wiley. Adapted with permission from Martins et al. (2017).

Gold nanoparticles were successfully supported on Al_2_O_3_, Fe_2_O_3_, ZnO, and TiO_2_ by Carabineiro and co-authors by DP (Martins et al., 2017). Their catalytic activity was assessed for the oxidation of cyclohexane at 60°C, atmospheric pressure, using H_2_O_2_ or TBHP (environmentally friendly oxidants). The results showed that Au nanoparticles were very active with no traces of by-products being detected under optimized conditions. Au/Al_2_O_3_ was the least active material (1.3% yield with TBHP), possibly due to its lower reducibility (shown by TPR). The yields achieved with Au/TiO_2_ and Au/ZnO were 4.0 and 3.2%, respectively (with TBHP). However, Au on Fe_2_O_3_ (with H_2_O_2_) showed a yield of 13.5% and was the best result obtained. It seems that, under those conditions, Au^+^ initial oxidation state (shown by Au/Fe_2_O_3_ and Au/TiO_2_) was more suitable for the oxidation of cyclohexane than Au^0^ (found on Au/ZnO and Au/Al_2_O_3_), as it lead to higher yields of cyclohexanol and cyclohexanone, in shorter reaction times. Notably, these yields were obtained at 60°C and atmospheric pressure, with low catalyst loads (Au catalyst to substrate molar ratio = 4 ×10^−3^), not requiring the presence of acid (as it was an inhibitor for these systems), being this more environmentally friendly. This system showed an almost exclusive unusual cyclohexanol formation by control of the reaction time (4 h). Catalyst recycling showed that the material was able to maintain high activity for 3 cycles (Figure 6), with not much leaching. The loss of activity shown for the 5th cycle is most likely due to adsorbed species on the surface, as shown by thermogravimetric experiments.

It has been controversially debated if Au acts as catalyst or as promotor of the oxidation reaction (Della Pina et al., 2012). Some authors believe that gold behaved as a real catalyst, as 10% conversion and 90% selectivity were obtained for with Au/TiO_2_-SiO_2_, but not on the support with no gold (Xu et al., 2007b, 2008). Nevertheless, Hereijgers and Weckhuysen studied the same reaction on Au/SBA-15, Au/Al_2_O_3_, and Au/TiO_2_, concluding that it follows a pure radical pathway with typical autoxidation products, being fully inhibited when radical scavengers are present (Hereijgers and Weckhuysen, 2010). However, Liu et al. demonstrated that gold/hydroxyapatite had high activity and that no reaction occurred in the support with no gold, although radical initiators, like TBHP, were needed (Liu et al., 2011). Hutchings and co-workers, when using Au/MgO catalysts, suggested an intermediate scenario, that is, Au could indeed accelerate the reaction and not need initiators (thus behaving like a real catalyst), but the acceleration took place when the amount of some species were increased (C_6_H_11_-OOH or C_5_H_11_-OO^•^), which promoted the catalytic processes by a radical chain mechanism (Conte et al., 2012).

**Scheme 12:**
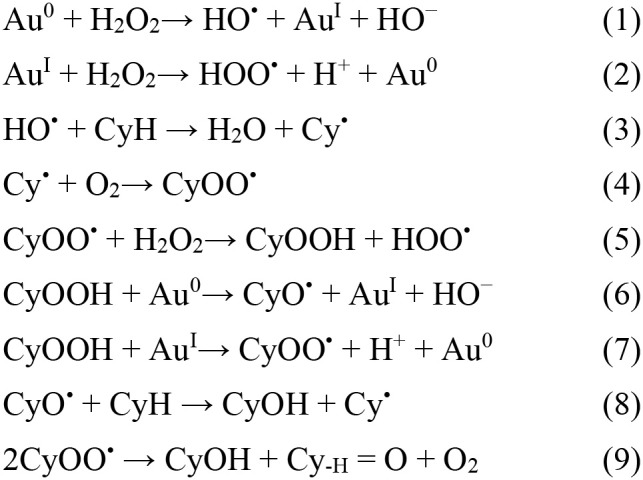
Proposed reaction mechanism or oxidation of cyclohexane to cyclohexanol and cyclohexanone in supported gold nanoparticles. Reprinted from Carabineiro et al. (2013). Copyright (2013), with permission from Elsevier.

Several authors report that the oxidation of cyclohexane by H_2_O_2_, catalyzed by metallic systems, proceeds mainly though a radical mechanism involving both C- and O-centered radicals (Alegria et al., 2007; Silva et al., 2008, 2009, 2010, 2011, 2013). Therefore, by analogy with the proposed mechanisms for several metallic systems (like Cu, Fe, Re, V) (Shulpin et al., 1993; Shul'pin et al., 2004; Nizova et al., 2002; Kopylovich et al., 2003, 2011; Shul'pin, 2003; Tanase et al., 2005, 2008; Alegria et al., 2007; Kozlov et al., 2007; Mishra et al., 2008, 2009; Silva et al., 2008, 2009, 2010, 2011, 2013; Di Nicola et al., 2009; Fernandes et al., 2009, 2011; Kirillova et al., 2009b; Shul'pina et al., 2009; Nesterov et al., 2012), a metal-catalyzed (and pyrazine carboxylic acid-assisted) decomposition of H_2_O_2_ was proposed by Carabineiro et al. (2013), shown in Scheme 12, based on what was observed by other authors (Quintanilla et al., 2012). Water can catalyze H^+^-shift steps leading the formation of HO^•^ from H_2_O_2_ (Kirillova et al., 2009a,b, 2011; Kuznetsov and Pombeiro, 2009). As suggested by Hutchings and co-workers for Au/MgO (Conte et al., 2012), gold can increase the reaction rate due to an increase in CyOOH or CyOO^•^ species.

Bimetallic Au/Pd catalysts on carbon based materials showed inferior activity than gold on the same supports (Mayani et al., 2016), and the same happened on TiO_2_ supports (Alshammari et al., 2015, 2016). However, Au-Ag/TiO_2_ showed better activity than Au/TiO_2_ (Alshammari et al., 2015). Also Au-Pd nanoparticles (on MgO) showed a significant positive influence on the overall catalytic performance, inhibiting the production of unwanted by-products (Liu et al., 2015). Au-Ag alloy catalysts, with metal nanoparticles immobilized on mesoporous silica, were also used (Wu et al., 2016a). A high catalytic activity and a high KA oil selectivity (>95%) were observed, due to the electronic structure modification caused by the synergistic effect of Au-Ag alloy nanoparticles.

### Oxidation of Other Alkanes

Gold catalysts have also been used also in *other alkane* oxidation reactions. As an example, Au on SiO_2_, SBA-15, Al_2_O_3_, ZSM-5, TiO_2_, ZrO_2_, CeO_2_, and Nb_2_O_5_ catalysts were used for the selective oxidation of *methane* to methanol with O_2_ (Hereijgers and Weckhuysen, 2011). Although all of them showed small gold nanoparticles, for temperatures above 250°C, very low activity was found, showing they were not active for this reaction. However, other authors (Kulikova and Shestakov, 2008) showed that Au nanoparticles, stabilized by a 1-dodecanethiol monolayer, were able to oxidize methane in a dichloromethane medium, to originate methanol and ethane. Au/SiO_2_ was also used to oxidize methane in ionic liquids, using K_2_S_2_O_8_ as oxidant, at 90°C, to produce methanol (Li T. et al., 2011).

Au-Ba/TS-1 catalysts (0.11% Au, Ti:Si ratio = 3:100) were found to be very selective toward the formation of acetone (90%), isopropanol (5%), and CO_2_ (5%) from *propane*, O_2_ and H_2_, at 170°C, after 0.5 h (Bravo-Suarez et al., 2008). Biradar and Asefa showed that SBA-15 supported Au nanoparticles were able to oxidize *n-hexane* (Biradar and Asefa, 2012). A 95% conversion, with a 92% selectivity to 2-hexanone and 8% selectivity to 2-hexanol, at 70°C, after 8 h reaction, using with TBHP as oxidant, were obtained (Biradar and Asefa, 2012). Larger alkanes are more difficult to oxidize, as shown by Hutchings and co-workers, that obtained very low conversion (~1%) in the oxidation of *n-decane*, with azobisisobutyronitrile, at 90°C, with 1.2 MPa O_2_, using a Au/ceria catalyst, even for 20 h reaction (Lloyd et al., 2011).

Recently, the aerobic oxidation of *several alkanes* (cyclohexane, propane, ethane and methane) to the corresponding alcohols, over Au_55_ (~1 nm size) nanoparticles with icosahedron symmetry, was investigated using density functional theory calculations (Staykov et al., 2018). Authors estimated that alkane hydroxylation proceeds through a two-step radical reaction mechanism. First, a hydrogen atom is abstracted from the alkane yielding a surface hydroxyl group and an alkyl radical. Then a reaction between the alkyl radical and hydroxyl radical takes place on the gold surface, being the rate limiting step for the overall oxidation.

## Conclusions

The obvious conclusion is that gold catalysts are very efficient for alcohol and alkane oxidation. The gold nanoparticle size and type of support continues to play a critical role, with smaller nanoparticles being more active, as in many other reactions. Gold has shown to be more active and selective than other noble metal catalysts. Gold on carbon is a very good catalyst for several reactions, but also gold on reducible oxides. However, composite supports and bimetallic gold catalysts are now emerging as new promising materials.

Looking into the future, gold nanoparticles have the potential to become very active catalysts leading to potential application of these reactions in industry, allowing them to occur in much milder and “greener” conditions. Nevertheless, the issue of durability might hinder such applications. The use of composite materials might be the way to overcome these challenges and obtain more active, selective and durable materials with potential industrial importance.

## Author's Note

Dedicated to Prof. Armando Pombeiro on the occasion of his 70th birthday.

## Author Contributions

SC did the literature search and wrote the paper.

### Conflict of Interest

The author declares that the research was conducted in the absence of any commercial or financial relationships that could be construed as a potential conflict of interest.
